# The Microbiome of Leonardo da Vinci’s Drawings: A Bio-Archive of Their History

**DOI:** 10.3389/fmicb.2020.593401

**Published:** 2020-11-20

**Authors:** Guadalupe Piñar, Maria Carla Sclocchi, Flavia Pinzari, Piero Colaizzi, Alexandra Graf, Maria Letizia Sebastiani, Katja Sterflinger

**Affiliations:** ^1^Department of Biotechnology, Institute of Microbiology and Microbial Biotechnology, University of Natural Resources and Life Sciences (BOKU), Vienna, Austria; ^2^Laboratorio di Biologia, Istituto Centrale per la Patologia degli Archivi e del Libro (ICPAL), Rome, Italy; ^3^Institute for Biological Systems (ISB), Council of National Research of Italy (CNR), Monterotondo, Italy; ^4^Applied Life Sciences/Bioengineering/Bioinformatics, FH Campus, Vienna, Austria

**Keywords:** third generation sequencing, nanopore technology, biological diagnosis, paper material, microbiome, Leonardo da Vinci, insect droppings, bio-archive

## Abstract

Seven emblematic Leonardo da Vinci’s drawings were investigated through third generation sequencing technology (Nanopore). In addition, SEM analyses were carried out to acquire photographic documentation and to infer the nature of the micro-objects removed from the surface of the drawings. The Nanopore generated microbiomes can be used as a “bio-archive” of the drawings, offering a kind of fingerprint for current and future biological comparisons. This information might help to create a biological catalog of the drawings (cataloging), a microbiome-fingerprint for each single analyzed drawing, as a reference dataset for future studies (monitoring) and last but not least a bio-archive of the history of each single object (added value). Results showed a relatively high contamination with human DNA and a surprising dominance of bacteria over fungi. However, it was possible to identify typical bacteria of the human microbiome, which are mere contaminants introduced by handling of the drawings as well as other microorganisms that seem to have been introduced through vectors, such as insects and their droppings, visible through the SEM analyses. All drawings showed very specific bio-archives, but a core microbiome of bacteria and fungi that are repeatedly found in this type of material as true degraders were identified, such as members of the phyla *Proteobacteria*, *Actinobacteria*, and *Firmicutes* among bacteria, and fungi belonging to the classes *Sordariomycetes* and *Eurotiomycetes*. In addition, some similarities were observed that could be influenced by their geographical location (Rome or Turin), indicating the influence of this factor and denoting the importance of environmental and storage conditions on the specific microbiomes.

## Introduction

Our cultural heritage is an asset that we must protect and monitor in order to pass it on to our future generations. One of the greatest pleasures is its contemplation in various permanent or temporary exhibitions. Most valuable graphic art pieces are displayed or stored in a particular museum, library or exhibition site under optimal conditions for their conservation and safety. However, it is common for these objects to be loaned out for certain thematic exhibitions in other museums or exhibition halls around the world. In this context, it is necessary to devise a method to monitor the risks of microbial contamination that may occur during packaging, transport and/or exhibition at the other sites and to allow a comparison before and after these trips. This monitoring is carried out by restorers and experts from the various institutions that provide and receive the object in question, but also by experts from renowned third-party institutions, which apply conventional technologies such as visual inspection, microscopy, but also introducing molecular technologies in order to achieve a detailed assessment of the risk of biological contamination posed by this type of transfer.

Conservators have long since started using new technologies such as miniaturized environmental condition recorders that keep track of how an object is stored during a move or an exhibition, away from the usual storage environment (Valentini et al., 2018). In addition to temperature and humidity monitoring systems, modern methods of classification and identification have also been developed, such as smart labels and barcodes technologies, which make it possible to quickly catalog objects and are already widely used in libraries. For example, the Radio-Frequency Identification systems (Steinberg et al., 2014) leverage radio waves to transmit data from chips to the readers (Alwadi et al., 2017). These systems help to cover traditional work processes such as check-in, check-out, anti-theft control and management of inventories. Even more innovative are the labels based on synthetic DNA (Demidov, 2020). These are patented systems that make it possible to make the art pieces not forgeable. Artists can authenticate their work by labeling it with a univocal synthetic bioengineered DNA sequence that provides an encrypted link between the artwork and a secure database containing the definitive information about the artwork. DNA data can be retrieved by sequencing or using other technologies like fluorescent probes (Banerjee et al., 2016). However, any object already contains DNA, from the people who have handled the object since its creation and from all the organisms and microorganisms that have come into contact with it, also through the deposition of dust and dirt. This natural bio-archive changes with time and can give certain information on some particular events, such as the presence on the materials of deteriorating microorganisms harmful for conservation, but also the permanence of the object in particular geographical locations, as indicated by the molecular traces left by the pollen of endemic plants (Piñar et al., 2019).

Molecular techniques, including DNA-based fingerprinting techniques in addition to cloning and Sanger sequencing, have been largely applied to investigate and monitor the biological colonization on art objects since more than two decades (Schabereiter-Gurtner et al., 2001). This molecular strategy, together with imaging and chemical analyses, has delivered relevant information about the microbial communities associated with different materials and the microbiological risk they represented on their surface (Sterflinger and Piñar, 2013). Nevertheless, the use of first-generation sequencing technology delivers limited data and is time consuming as well as not free of bias. This strategy has become obsolete and has been replaced by new high-throughput molecular technologies in which massive DNA sequencing is possible, offering a much broader view of the real communities that colonize our cultural heritage. Next Generation Sequencing (NGS) technologies have evolved incorporating revolutionary improvements to address the complexities of genomes and metagenomes at an unprecedented speed (Goodwin et al., 2016), being now well established in the field of cultural heritage. These analyses can be performed either by the so-called “shotgun metagenomic approach,” by sequencing the entire DNA library representing all the molecular components of a given sample (Teasdale et al., 2017; Piñar et al., 2020b), or by focusing on specific conserved sequences such as ribosomal RNA genes (Marvasi et al., 2019). The latter approach called “target amplification approach” has some advantages, such as the reduction of data complexity, the possibility to assign more sequences to specific organisms and finally, facilitates some semi-quantitative analysis. This approach is extremely useful in the field of cultural heritage, as DNA with a poor quality or low concentration can be amplified by using degenerate primers and PCR. Many of the studies using the latter strategy have been collected and summarized in the review of Marvasi et al. (2019). These methods are powerful, but as in any PCR-based method, primers and/or exponential amplification introduce bias. In addition, the simultaneous quantitative assessment of all three domains of life is impossible. Furthermore, the amplification of target sequences (mainly ribosomal sequences) do not allow us to draw conclusions on any functional genes and therefore the microbial functional traits remain uncertain. Nevertheless, the further technological development of molecular methods is overcoming many technical biases. Third generation sequencing technologies are emerging and offering some advantages over the limitations suffered by NGS platforms, such as the short-read length that needs to be assembled with the help of bioinformatic tools into the original length template, and the PCR bias introduced by clonal amplification for the detection of base incorporation signal.

Third generation sequencing technologies offer an intelligent solution to the limitations mentioned above (Schadt et al., 2010). Instead of sequencing a clonally amplified template, a single DNA template is sequenced, leading to a reduced use of reagents and chemicals, simplified protocols, and miniaturization of the entire technical process and equipment. One example is the Nanopore sequencing technology, which involves the use of protein nanopores embedded in a polymer membrane. The sequencing does not necessarily require any intervening PCR amplification or chemical labeling step. Furthermore, this technology offers a pocket-sized sensing device, the MinION, which offers the advantages of a portable device. To date, the Nanopore sequencing technology has been scarcely applied in the field of cultural heritage, and only four studies have reported on the advantages of applying this state-of-the-art technology on valuable art objects. Three of these studies (Šoltys et al., 2019; Grottoli et al., 2020; Kisová et al., 2020) reported on the advantages of sequencing long DNA fragments when targeting ribosomal regions. However, all three studies were based on the target amplification approach that avoided inferring the relative proportion of the different phylogenetic groups. The fourth study (Piñar et al., 2020a) applied for the first time the Nanopore sequencing technology together with a whole genome amplification (WGA) protocol to perform a rapid diagnosis of the biological infection on objects of art, unifying all the advantages that this new technology can offer for metagenomic analyses starting from very low DNA concentrations and reflecting the real proportions of all domains of life.

Although molecular techniques are not completely free from bias (Sterflinger et al., 2018a), the establishment of next- and third-generation sequencing technologies in the field of cultural heritage is providing a powerful DNA-based approach to monitor the preservation status as well as to foresee the risk of deterioration of valuable objects (Piñar et al., 2020a). The data generated can provide interesting information that can contribute to surprising insights into the objects being investigated, such as the selection of materials at the time of manufacture and the conditions of their storage or about possible displacements and geographical origins, but also uncover important information about the object’s history of use (Teasdale et al., 2017; Piñar et al., 2019, 2020b). This information may help to understand many open questions in a variety of fields, as archeology, history, restoration, philology, criminology but also, they can contribute to giving an historical benefit to the investigated objects in form of a bio-archive (Piñar et al., 2019, 2020b).

In the present study we apply the Nanopore sequencing technology in combination with a whole genome amplification (WGA) protocol to survey some of Leonardo da Vinci’s most emblematic drawings. This strategy was applied as the only diagnostic method or in combination with SEM analyses, in order to explore the nature of contaminants deposited on the surfaces of the drawings. The sampling strategy, based on a very delicate micro-aspiration, was aimed primarily at collecting dust particles, microbial cells and other debris from small areas of surfaces. The aim was to create a bio-archive of the dust and other collectable material deposited on the drawings, in order to monitor their conservation status and thus be able to use them for possible comparisons in the future. The sampling was not used to assess the percentage of living propagules, however, the molecular strategy adopted could theoretically indicate the presence of fungal or bacterial cells with an anomalous abundance, compared to typical indoor dust composition. Furthermore, the information contained in this bio-archive is an added value for the drawings, that can in turn help to understand some issues related to the history of storage of the investigated drawings. This study represents one more example of how the Nanopore technology, in this case in combination with microscopy techniques, can be a practical tool for the rapid biological diagnosis and monitoring in priceless objects.

## Materials and Methods

### Objects and Sampling

**FIGURE 1:**
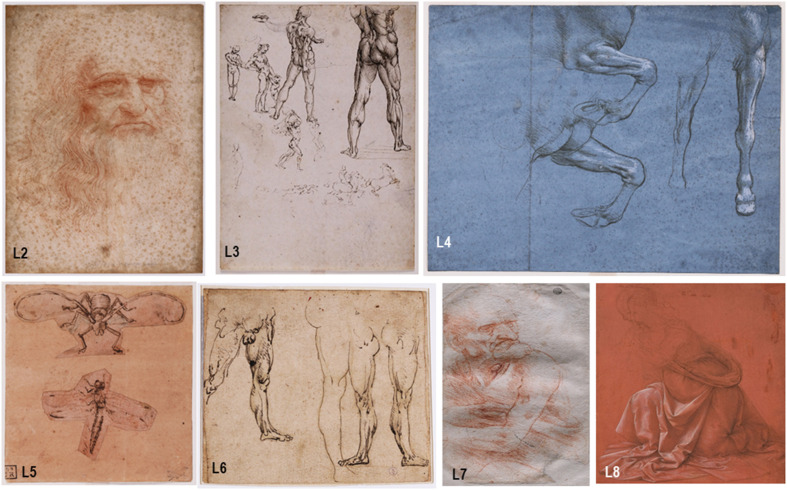
The Leonardo da Vinci’s drawings investigated. The drawings currently housed in the Royal Library of Turin are: “*Autoritratto*” (sample L2, 33.3 × 21.3 cm), “*Nudi per la battaglia di Anghiari*” (sample L3, 25.4 × 19.7 cm), “*Studi delle gambe anteriori di un cavallo*” (sample L4, 15.4 × 20.5 cm), “*Studi di insetti*” (sample L5, 12.9 × 11.8 cm) and “*Studi di gambe virili*” recto, “*Figura presso il fuoco*” verso (sample L6, 14.0 × 15.7 cm). The drawings stored at the Corsinian Library in Rome are: “*Uomo della Bitta*” (sample L7, 22 × 14.6 cm) and “*Studio di panneggio per una figura inginocchiata*” (sample L8, 25.7 × 19.0 cm).

Seven emblematic drawings from Leonardo da Vinci were selected for the analysis of their microbiomes (Figure 1). Five of these drawings are currently housed in the Royal Library of Turin, located on the ground floor of the Royal Palace in Turin (Italy): “*Autoritratto*” (sample L2, 33.3 × 21.3 cm), “*Nudi per la battaglia di Anghiari*” (sample L3, 25.4 × 19.7 cm), “*Studi delle gambe anteriori di un cavallo*” (sample L4, 15.4 × 20.5 cm), “*Studi di insetti*” (sample L5, 12.9 × 11.8 cm) and “*Studi di gambe virili*” *recto*, “*Figura presso il fuoco*” *verso* (sample L6, 14.0 × 15.7 cm). The last two investigated drawings are stored at the Corsinian Library in Rome (Italy): “*Uomo della Bitta*” (sample L7, 22 × 14.6 cm) and “*Studio di panneggio per una figura inginocchiata*” (sample L8, 25.7 × 19.0 cm).

Conservators of the ICPAL (Istituto Centrale per la Patologia degli Archivi e del Libro) collected samples from the *recto* and the *verso* of the selected drawings. A micro-sampling method for fragile surfaces has been specifically developed based on a protocol tested mainly on paintings and described by de Carvalho et al. (2019). The system is based on a micro-aspiration aimed primarily at collecting dust particles, and microbial cells from small areas of paper or parchment surfaces (Supplementary Figure 1). The suction force applied was modulable, and the filter membranes used to trap the particulates were sterile and made of a material suitable for subsequent use in both molecular and SEM analysis. The micro-aspiration system was also used to sample the microscopic objects detached from the drawings with needles under an optical microscope, as in the case of single fibers or particles of a few hundred microns. The suction system was an autoclavable polypropylene filter holder, 13 mm diameter (Swinnex filter holder, Millipore) with sterile membranes (Millipore cellulose nitrate membrane filter Ø = 13 mm, Pore size = 0.45 μm) connected to an oil-free diaphragm vacuum pump (DA14 Charles Austen MB2238. Appleton Woods Ltd., United Kingdom).

The total number of membranes used for sampling varied between the drawings, ranging from 10 to 36 membranes per drawing (depending on the size of the drawing). Each single used membrane was immediately introduced in a single sterile Eppendorf tube and appropriately labeled for the storage of the samples until DNA extraction analyses. The same kind of membranes was used for SEM observation of the particulate sampled from the drawings and its chemical analysis.

### DNA Extraction

The DNA was extracted using the FastDNA SPIN Kit for soil (MP Biomedicals, Illkrich, France) as recommended by the manufacturers. DNA extraction was performed directly from the membranes by grouping a maximum of four membranes per reaction tube. DNA extracted from each single reaction tube was pooled per sample to obtain a single microbiome from each drawing. The DNA concentrations were assessed by using the Qubit 2.0 fluorometer (Invitrogen Corporation), with the Qubit dsDNA HS Assay Kit.

### Whole Genome Amplification (WGA), Template Preparation and Sequencing

Whole genome amplification (WGA) and template preparation was performed following the “Premium whole genome amplification protocol” available in the Oxford Nanopore community. For WGA, all reactions were executed in a BioRad C1000 Thermal Cycler using the REPLI-g Midi Kit (Qiagen), which uses the innovative Multiple Displacement Amplification (MDA) technology. The libraries were performed using the Ligation Sequencing kit 1D SQK-LSK109 and the Flow cell Priming kit EXP-FLP001 (Oxford Nanopore Technologies). All steps performed for library preparation were performed as described by Piñar et al. (2020a). A quality control of flow cells (SpotOn Flow cell Mk I R9 Version, FLO-MIN 106D) was performed prior starting the sequencing. The MinKNOW^TM^ software was used to check the number of active pores in the flow cells (Table 1) and for performing the sequencing runs. All seven runs were performed for 48 h.

**TABLE 1:** Details of the sequencing runs performed on seven drawings of Leonardo da’Vinci using the MinIon (Nanopore sequencing technology).

**Drawing**	**Sample (ID run)**	**Active pores/flow cell**	**Total reads^a^**	**Analyzed reads^b^**	**Classified reads^*c*^**	**Total yield**	**QS^d^**	**Average length (Kb)**
“*Autoritratto*”	**L2** (193982)	1518	1,775,691	1,543,693	166,129	7.7 Gb	8.77	4.34
“*Nudi per la battaglia di Anghiari*”	**L3** (193283)	1639	1,354,462	1,168,117	123,072	5.0 Gb	9.26	3.71
“*Studi delle gambe anteriori di un cavallo*”	**L4** (193072)	1706	2,796,008	2,408,671	1,009,200	12.4 Gb	9.08	4.44
“*Studi di insetti*”	**L5** (193067)	1558	2,857,065	2,461,722	317,761	13.9 Gb	9.09	4.86
“*Studi di gambe virili*”/*“Figura presso il fuoco”*	**L6** (192071)	1570	3,168,743	2,796,829	414,016	16.8 Gb	9.51	5.31
“*Uomo della Bitta*”	**L7** (202641)	1512	1,710,990	1,514,255	65,343	6.8 Gb	10.94	3.97
“*Studio di panneggio per una figura inginocchiata*”	**L8** (203053)	1523	2,732,413	2,376,913	183,811	14.2 Gb	10.38	5.19

*^a^Total reads: Total reads generated in the sequencing run. ^*b*^Analyzed reads: Total number of reads processed by WIMP. ^*c*^Classified reads: Number of reads classified phylogenetically. ^*d*^QS, Quality Score.*

### Data Analyses

Resulting fast5 data files were basecalled using the Nanopore GPU basecalling with GUPPY 3.0.3 on UBUNTU 16.04 (Nanopore Community Platform). Once the Fastq files were generated, the data were compared with databases using one of the available pipelines for data analyses of the Nanopore Community Platform, following the steps recommended by manufacturers. The selected workflow chosen was “What’s in my pot” (WIMP), which is an EPI2ME workflow for taxonomic classification of basecalled sequences (reads) generated by Nanopore sequencing. WIMP initially filters FASTQ files with a mean q-score below a minimum threshold (defaults to 7).

Relative abundances, taxonomic clustering, correspondence analysis and alpha diversity analysis were performed using the R packages taxonomic, phyloseq and microbiome for the R version 4.0.0. (McMurdie and Holmes, 2013). Before microbial taxonomic clustering was performed human reads where removed and relative abundances were calculated.

### Nucleotide Sequence Accession Number

The sequences obtained by the metagenomic analysis are deposited and publicly available under the BioProject ID: PRJNA655381

### Optical Microscopy

Features of interest in the drawings were documented with a Leica MZ16 stereoscopic microscope fitted with low-temperature optic fiber lights. The system was equipped with a digital camera connected with a computer and software (Leica Application Suite, LAS, Leica Microsystems GmbH Wetzlar, Germany) that allowed the acquisition of images at different magnifications.

### Scanning Electron Microscopy and Microanalysis

The micro-objects removed from the drawings, fibers and membranes used to sample the drawings were analyzed using a variable pressure SEM instrument (EVO50, Carl-Zeiss Electron Microscopy Group) equipped with a detector for backscattered electron diffraction (BSD). After SEM imaging using Variable Pressure mode, some of the samples were covered with gold for further analysis in High Vacuum mode.

The samples were placed on a sulfur-free carbon adhesive (Spectrotabs AGG3358, Agar Scientific) glued onto an aluminum stub (1.25 cm diameter, Agar Scientific). Chemical analysis was performed by means of EDS (INCA 250, Oxford Instruments). The SEM was fitted with a tungsten filament and operated at 20 keV, with an average working distance of 12.5 mm, with a chamber pressure between 30 and 150 Pa, chosen to keep unaltered micro-structures. The electron beam current was adjusted (∼400 pA) to generate sufficient X-rays to allow reliable identification of EDS peaks, minimize spectral artifacts and achieve detector dead times < 20%. The beam spot size was 5–30 nm in diameter. Acquisition live time for single analysis was set at 50 s. We performed EDS analyses of several types. Some were point analyses, and some consisted of the approximate entire surface of fibers and particles. Conventional ZAF correction (for atomic number Z, absorption and fluorescence) was applied, integrated into the Oxford INCA 250 microanalysis software used.

### Statistical Analysis of EDS Chemical Data

The EDS measurements were analyzed using statistical tests to evaluate the relationships between variables and the significance of the differences between samples. One-way analysis of the variance (ANOVA) was applied and the significance of the differences was tested at 95% confidence. The ANOVA model used was “unbalanced” because the number of observations within each category was not the same. ANOVA was followed by a *post-hoc* analysis using Tukey’s *t*-test to determine the critical value for significance (McHugh, 2011). The measure of linear dependence (correlation) between couples of analyzed elements from the EDS datasets was obtained using the Pearson’s coefficient ”r. This coefficient has a value between +1 and −1, where +1 is total positive linear correlation, 0 is no linear correlation, and −1 is total negative linear correlation. The *p*-values that were computed for each coefficient allowed testing the null hypothesis that the coefficients were not significantly different from 0 (Reimann et al., 2008). ANOVA, PCA and Pearson’s correlation coefficient analyses were performed using XLSTAT 2017 software (Addinsoft, Paris, France).

## Results

### DNA Yield and Sequencing Analyses

Seven outstanding drawings by Leonardo da Vinci were investigated (Figure 1) by using non-invasive sampling with suction membranes (Supplementary Figure 1), due to the strict policies and requirements for obtaining samples of the invaluable objects under investigation. The DNA was directly extracted from the membranes collected from each drawing and pooled, as explained in the materials and methods section. Results showed an expected low concentration of DNA, ranging from 5.4 to 20 pg/μl and for this reason, a protocol including a WGA, available in the Oxford Nanopore community was chosen (Piñar et al., 2020a), which enables to prepare DNA libraries with as little as 10 pg total DNA as input. Seven independent sequencing runs were carried out, each in an independent Nanopore flow cell loaded with the DNA library prepared from each of the drawings. Table 1 presents the details of the sequencing runs performed with the Nanopore MinION device, showing an average of 2,342,196 total reads generated per sequencing run. The reads analyzed and subsequently classified by the WIMP workflow showed an average of 2,038,600 and 325,619, respectively, indicating that about 16% of the analyzed reads were phylogenetically affiliated. In addition, the total yield obtained in the seven sequencing runs showed an average of 11 Gb, the average length of the fragments sequenced was 4.5 Kb, and the quality score (QS) showed an average of 9.6.

### Microbiomes Generated From Each Drawing

**FIGURE 2:**
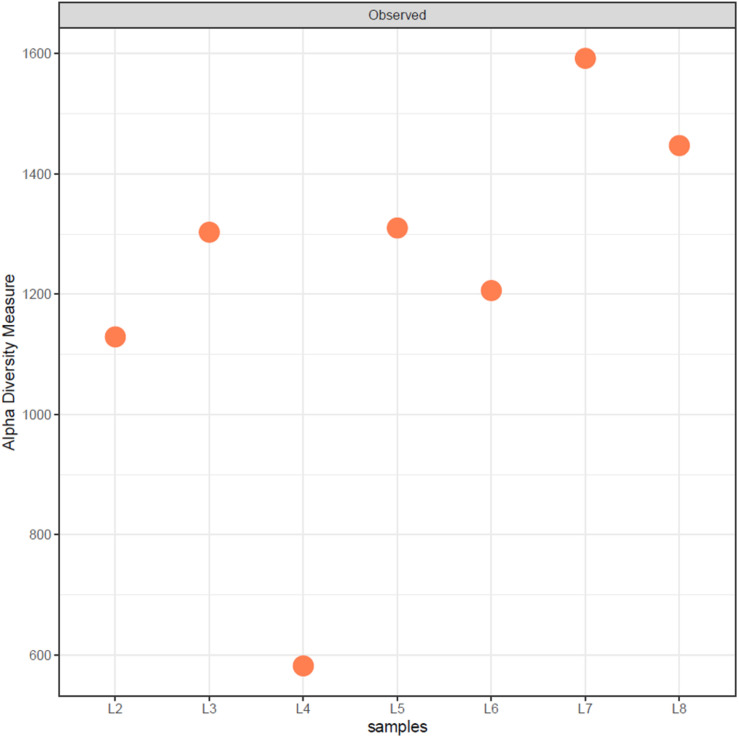
Alpha diversity of drawings calculated from the rarefied classified abundance data using the R package phyloseq.

**FIGURE 3:**
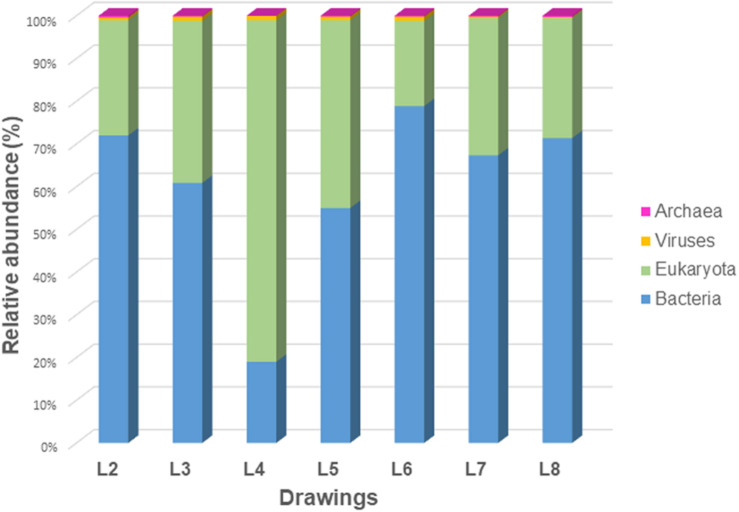
Relative abundance of eukaryotes, bacteria, archaea, and viruses in the microbiomes of all seven investigated drawings.

The results showed that the proportion of the analyzed reads that could finally be phylogenetically assigned using the Nanopore WIMP workflow was different for each drawing (Table 1). However, there was an overall high biodiversity in the microbiome of the drawings investigated, revealing differences but also intriguing similarities between some of them. Drawings L7 and L8 showed the highest biodiversity, followed by drawings L5, L3, L6, and L2, all with similar alpha diversity, while L4 showed the lowest diversity of all (Figure 2). The percentage of human reads varied between 5.35% (L2) and 74.64% (L4) of the classified reads with a median of 11.36%. Therefore, we can assume that the high number of human reads recovered from the surface of drawing L4 masked the detection of reads related to microorganisms in this drawing. Figure 3 shows the distribution of the different superkingdoms among the investigated drawings. The relative abundance of bacteria ranged from 19% (with lowest proportion in drawing L4) to 79% (with highest proportion in drawing L6). Eukaryotes ranged from 20 to 80%, being the highest proportion present in drawing L4, once more mainly due to the massive presence of human DNA in this drawing. Viruses and Archaea represented less than 1% of the total microbiome in all investigated drawings.

**FIGURE 4:**
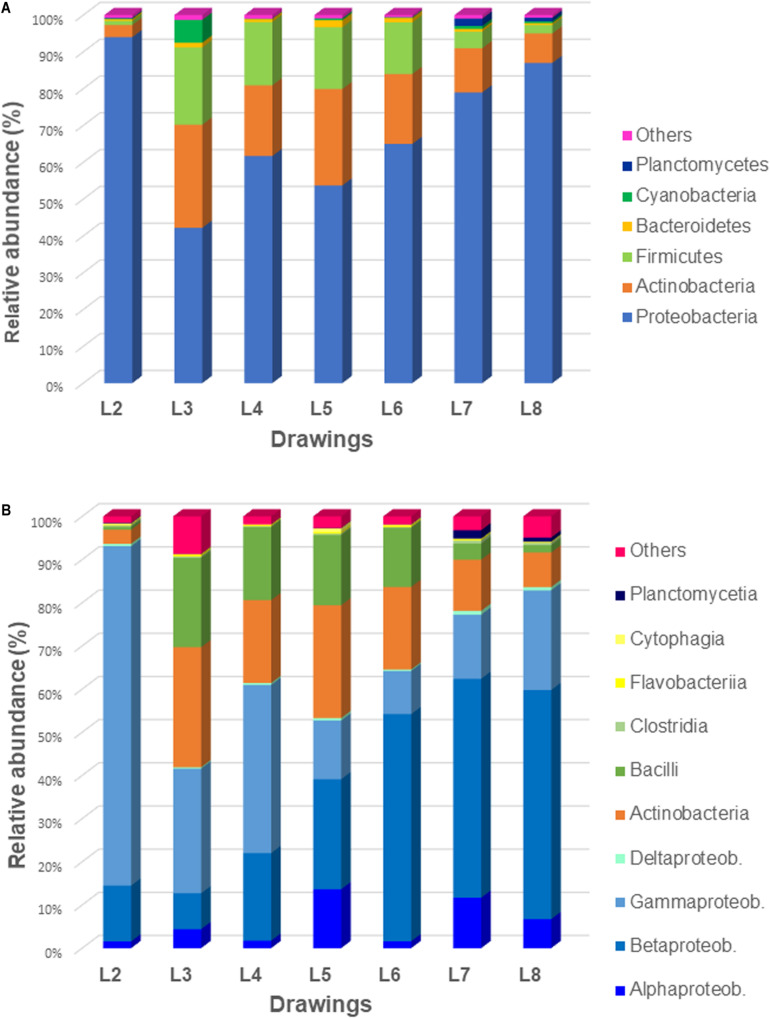
Relative abundance of the bacterial communities of all seven investigated drawings: **(A)** at the phylum level and **(B)** at the class level.

**FIGURE 5:**
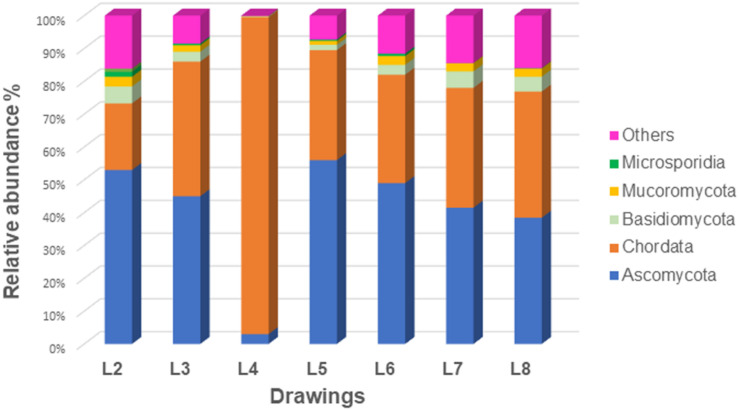
Relative abundance of the eukaryotic communities of all seven investigated drawings at the phylum level.

Concerning bacteria (Figure 4A), the phylum *Proteobacteria* dominated in all samples (42–94% of bacteria), followed by members of *Actinobacteria* (3–28% of bacteria) and *Firmicutes* (1–21% of bacteria). The phylum *Cyanobacteria* showed to be represented only in the drawing L3 (6% of bacteria) while the phylum *Planctomycetes* was represented only in drawings L7 and L8 (2 and 1% of bacteria, respectively). The Eukaryotes (Figure 5) were mainly represented by the phylum *Ascomycota* (3–56% of eukaryotes) with the exception of the microbiome of drawing L4, which showed a massive dominance of the phylum *Chordata* (over 96% of the eukaryotes). This last phylum showed to be well represented in all drawings due to the presence of human DNA. Members of the phyla *Basidiomycota*, *Mucoromycota*, and *Microsporidia* were identified in all the drawings with relatively low proportions (0.5–5% of eukaryotes), in addition to some other phyla, each representing less than 0.5% of the microbiomes (marked as “others” in Figure 5).

**FIGURE 6:**
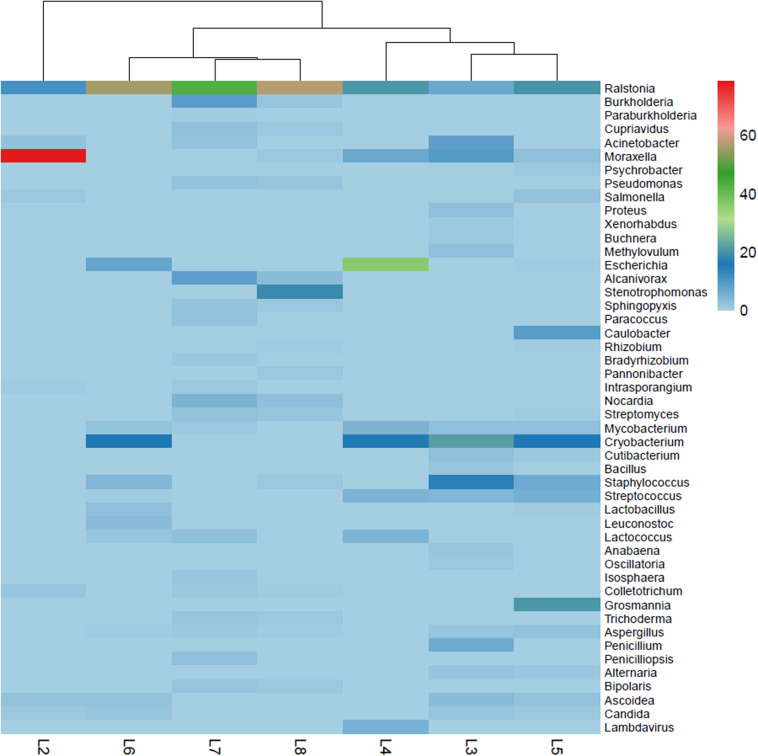
Heatmap reporting the relative abundance (%) of genera in each of the investigated drawings; the genera shown represent greater than 0.5% abundance in at least one sample.

Each drawing owned a very specific microbiome, showing an independent molecular profile or “biological pedigree.” Supplementary Figure 2 shows all genera that represent more than 0.5% of the total microbiome in each of the drawings (cutoff 0.5%). These biological pedigrees are all summarized in Figure 6 in form of a heatmap, showing the relative abundance (%) of each genus in each of the drawings.

#### Bacterial Communities

The bacterial communities dominated over the eukaryotic communities in all the drawings. The most dominant phylum showed to be the *Proteobacteria* with dominance of the *Gammaproteobacteria* class in drawings 2–4 (Figure 4B). Inside this class, members of the order *Pseudomonadales* were detected, such as the genus *Acinetobacter*, present in drawings L2, L3, and L7, the genus *Moraxella*, which was highly abundant in L2 (representing over 51% of total reads) but also relative abundant in L3–L5 (1–5.25% total reads), the genus *Psychrobacter* being representative solely in L5 and the genus *Pseudomonas*, which was relative abundant in drawings L7 and L8 (Figure 6). Members of the *Enterobacteriales* were detected in drawings L2–L6 but absent in drawings L7–L8. The genus *Salmonella* was detected in L2 and L5 while *Escherichia* was identified in L4–L6. The genera *Proteus, Xenorhabdus* and *Buchnera* were present exclusively in L3 (Figure 6). Interestingly, the genus *Alcanivorax* (order *Oceanospirillales*) was present in drawings L7 and L8 and *Stenotrophomonas* (order *Xanthomonadales*) was only present in L8 with a high relative abundance (over 10% of total reads, Figure 6).

In contrast, members of the *Betaproteobacteria* class were shown to be more abundant in drawings L5–L8 (Figure 4B) with *Ralstonia* as the dominant genus in all drawings (∼3–40% of total reads) (Figure 6). The genera *Burkholderia* and *Cupriavidus* were significative only in drawings L7 and L8 and *Paraburkholderia* exclusively in drawing L7. Members of the *Alphaproteobacteria* class were identified in all drawing but in relative lower proportions (Figure 4B). The genus *Caulobacter* showed to be exclusively present and with a relative high abundance (over 5% of total reads) in drawing L5. The genus *Rhizobium* was identified in L5 and L8 while *Bradyrhizobium* only in L8. The genus *Sphingopyxis* was shown to be present in L7 and L8, while *Paracoccus* was only identified in L7 and *Pannonibacter* only in L8 (Figure 6).

The Actinobacteria were the second most represented phylum within the bacteria, with all members identified belonging to the *Actinobacteria* class (Figure 4B). Within the order *Corynebacteriales*, the genus *Mycobacterium* was present in drawings L3–L7 while the genus *Nocardia* was only identified in L7 and L8. Members of the order *Micrococcales* were identified in almost all the drawings, being the genus *Intrasporangium* only present in L2 and L7 and the genus *Cryobacterium* widely distributed among L3–L6. In addition, the genus *Streptomyces* (order *Streptomycetales*) was well represented in drawings L7 and L8 but also in lower proportion in L5 while *Cutibacterium* (order *Propionibacteriales*) was identified in L3 and L5 (Figure 6).

The phylum Firmicutes was mainly represented by members of the *Bacilli* class (Figure 4B), with genera of the order *Bacillales*, such as *Bacillus* present exclusively in L3 and *Staphylococcus*, present in a relative high abundance in L3 (over 8% of total reads) but also well represented in L5 and L6 (4 and 3.15% of total reads, respectively) and in lower proportion in L8 (Figure 6). Members of the order *Lactobacillales* showed to be widely represented in almost all drawings, with the genera *Streptococcus* (in drawings L3–L6), *Lactobacillus* (in drawings L5 and L6), *Lactococcus* (in L4, L6, and L7) and *Leuconostoc* (solely in L6). Members of the *Clostridia* class were detected (Figure 4B), but each of the identified genera contributed less than 0.5% of the total reads.

The same occurred with the *Flavobacteria* and *Cytophagia* classes within the phylum Bacteroidetes, no single genus accounted for more than 0.5% of the total reads (Figure 4B). The phylum Planctomycetes showed to be represented only in drawings L7 and L8 with members of the *Planctomycetia* class. Inside this class, only the genus *Isosphaera* accounted for more than 0.5% of the total reads in L7. Finally, the phylum Cyanobacteria was only significantly represented in L3 with the genera *Anabaena* and *Oscillatoria*, both accounting for more than 0.5% of total reads in this drawing (Figure 6).

#### Eukaryotic Communities

First of all, it is important to note that an average of 34% of the eukaryotic sequences that could be identified in the drawings showed to be affiliated with the *Chordata* phylum (Figure 5), being all related to human sequences, with the exception of drawing L4, where these sequences represented more than 96% of the eukaryotic sequences. The remaining eukaryotic sequences were affiliated with fungi distributed in different phyla, i.e., *Ascomycota* (3–56% of eukaryotes) and in lower proportions *Basidiomycota*, *Mucoromycota* and *Microsporidia* (0.5–5% of eukaryotes), in addition to some other phyla, each representing less than 0.5% of the microbiomes.

Only a few taxa of the phylum Ascomycota represented more than 0.5% of the total reads in the microbiomes of the drawings (Figure 6). Thus, members of the *Eurotiomycetes* class proved to be widely distributed among the drawings, with species of the genus *Aspergillus* identified in L3, L5–L8, while species of the genera *Penicillium* (in L3) and *Penicilliopsis* (L7) were representative in only one of the drawings. Members of the *Sordariomycetes* class of the genus *Colletotrichum* showed to be representative in L2, L7, and L8. The genus *Trichoderma* was representative only in the last two drawings (L7 and L8) while the genus *Grosmannia* was solely identified in L5 in a relatively high proportion (12.6% of total reads) (Figure 6). Within the *Dothideomycetes* class, species of the genus *Alternaria* were representative in drawings L3 and L5 and of the genus *Bipolaris* in L7 and L8. Finally, members of the *Saccharomycetes* class showed to be widespread in drawings L2, L3, L5, and L6, with species of the genera *Ascoidea* and *Candida* (Figure 6).

### Canonical Correspondence Analyses

**FIGURE 7:**
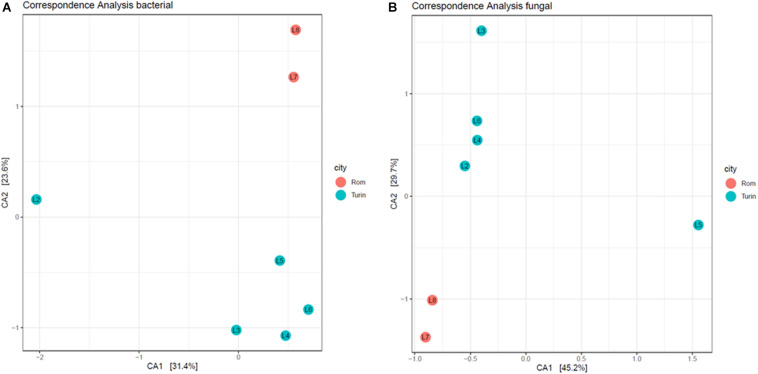
Canonical correspondence analysis plot of the first and second CA axis for bacterial **(A)** and fungal **(B)** abundance data. Dots represent samples (sample name is indicated within the dots) and colors represent the city where the drawings are located—Turin (blue) and Rom (red). Percentages indicate how much of the variability in the data is explained by the respective axis.

Canonical correspondence analysis was used as an ordination method, providing a spatial representation of the relationship of taxa and samples. In the first CA axis of the bacterial community (Figure 7A), the drawing L2 showed a strong separation from the other drawings, indicating that the bacterial community in this drawing differs significantly from that detected in the others, mainly due to the high impact of the genus *Moraxella* on this sample. Looking at the fungal composition (Figure 7B), the drawing L5 clustered distinctly from the others in the first CA axis, confirming the high impact of the fungus *Grosmannia* on the fungal community. In the second CA axis, the separation between samples from Turin and from Rome can be seen within fungal as well as bacterial species, indicating a geographical influence on the microbiome of the drawings and denoting the importance of environmental and storage conditions on the specific microbiomes.

### SEM Analyses

For obvious reasons of relevance, fragility and uniqueness of the objects studied, the microscopic analysis of the drawings was mainly aimed at acquiring photographic documentation using non-invasive techniques, using above all a stereoscopic microscope with fiber optic illumination. The nature of micro-objects that the restorers were able to remove from the surface without causing damage could then be explored further by SEM-EDS. These were mostly particles superimposed on the fibers without any apparent historical value. In addition, some of the membranes used to remove dust from the surface of the designs undergoing molecular investigation were analyzed with SEM-EDS mainly to assess the effectiveness of the sampling method applied.

**FIGURE 8:**
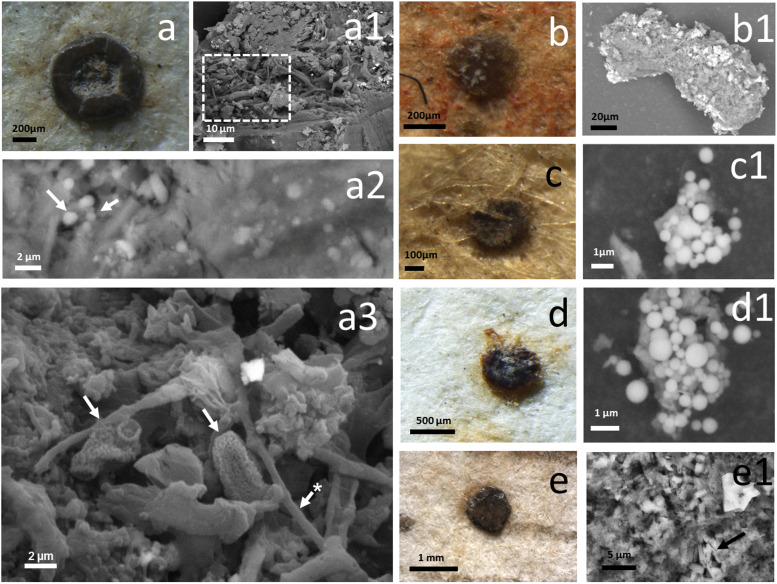
Insects’ droppings. Waxy brown incrustations documented on the fibers of drawing L4 (*Studi delle gambe anteriori di un cavallo*) and drawing L2 (*Autoritratto*). These “objects” have been identified as insects’ droppings. They have been documented and, where possible, removed and analyzed by SEM-EDS. **(a)** an incrustation that was defacing the verso of drawing L4; **(a1,a2)** SEM images of the waxy material. The images were obtained in variable pressure, with a backscattered detector on non-metallized samples. **(a1)** shows the appearance of the surface of the object, covered with fungal mycelium, the detail in the square frame corresponds to **(a3)**. The arrows in **(a2)** show round-shaped crystals embedded in the amorphous matrix. **(b,b1,c,c1,d,d1)** Correspond to incrustations that were present on the drawing L2. These were made both of an amorphous matrix, as in **(b1)**, but also by spherulites, that consist of spherical crystals made of calcium and magnesium phosphate. **(e,e1)** Show an incrustation of the L4 drawing where in the amorphous matrix were documented columnar and bipyramidal crystals (arrow).

On many of the drawings analyzed, roundish waxy brown incrustations were specifically observed, often surrounded by greasy looking halos on the underlying fibers. These recurrent “objects” have been documented and in some cases removed and chemically analyzed. Figure 8 shows the incrustations on L2 and L4 drawings, where they were particularly abundant, before their removal and their corresponding SEM images. The chemical analysis of three waxy incrustations removed from each of the two drawings is shown in Table 2, which shows the average values of a minimum of 10 EDS observations for each particle and the analysis of the statistical significance of the differences found between them. The EDS elemental analysis is not quantitative but rather qualitative. The values obtained are therefore mostly indicative. However, the comparison between the chemical composition of samples of similar nature and size, analyzed in the same way, may have a statistical value. Similarly, the correlation, and therefore the stoichiometric ratio, between pairs of chemical elements in each sample is significant because the technique measures the chemical composition of a small volume, in the order of nanometers, which therefore reflects the concentration of the elements and their relative percentage in that volume. In light of this, it is interesting what has emerged from the composition of the objects, which differ between the two drawings, and which contain magnesium phosphate in one case (L2 drawing) and potassium sulfate in the other (L4 drawing) (Supplementary Figure 3).

**TABLE 2:** EDS analysis of three insects’ droppings removed and analyzed from drawing L4 (“*Studi delle gambe anteriori di un cavallo”)* and drawing L2 (“*Autoritratto”).*

	**C**	**N**	**O**	**Na**	**Mg**	**P**	**S**	**K**	**Ca**	**Fe**
L4.1	75.41^a^	4.66^bc^	14.10^b^	0.70^a^	0.24^c^	0.65^b^	2.45^a^	1.32^b^	0.41^b^	0.06^b^
L4.2	67.68^b^	8.58^bc^	15.89^b^	0.51^b^	0.48^c^	1.25^b^	2.50^a^	2.10^a^	0.83^b^	0.18^ab^
L4.3	56.03^c^	23.96^a^	15.48^b^	0.38^c^	0.28^c^	0.62^b^	1.69^b^	1.12^b^	0.41^b^	0.04^b^
L2.1	57.35^c^	0.00^c^	31.23^a^	0.08^e^	1.13^a^	3.12^a^	0.86^c^	0.13^c^	5.63^a^	0.48^a^
L2.2	59.29^c^	0.00^c^	30.70^a^	0.20^d^	0.90^b^	3.31^a^	0.58^c^	0.00^c^	5.02^a^	0.00^b^
L2.3	54.49^c^	12.63^b^	26.44^a^	0.02^e^	0.43^c^	1.05^b^	0.51^c^	0.16^c^	3.89^a^	0.38^ab^
Pr > F (Model)	<0.0001	<0.0001	<0.0001	<0.0001	<0.0001	<0.0001	<0.0001	<0.0001	<0.0001	0.02
Significant	Yes	Yes	Yes	Yes	Yes	Yes	Yes	Yes	Yes	Yes

*Values are the means from a minimum of n = 15 replicates. In each column, different letter indicates statistically significant differences (P < 0.05), one-way ANOVA.*

**FIGURE 9:**
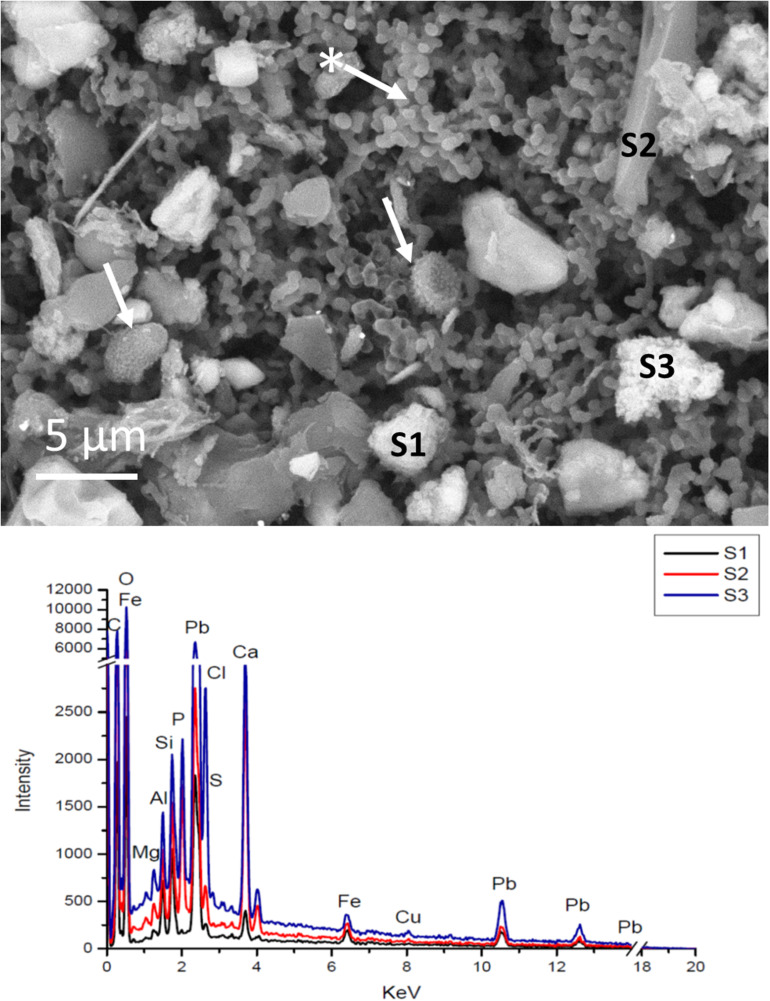
SEM images of the surface of a membrane used to sample the surface of the drawing L3 (*Nudi per la battaglia di Anghiari*). The image was obtained in variable pressure, with a backscattered detector on non-metallized samples. The arrow with the asterisk indicates the appearance of the membrane polymer itself. The other arrows indicate fungal spores (*Aspergillus* or *Penicillium*). The EDS spectra corresponding to the labeled particles are reported in the plot below. In this drawing several mineral particles were containing lead, silicon or calcium as the main element.

SEM imaging and EDS analysis of the membranes used for sampling the airborne material deposited on the drawings between the fibers and destined to outline their genetic “pedigree,” showed the effectiveness of the suction system which, without contact with the drawing, made it possible to recover significant micro-objects including fungal spores. Figure 9 shows SEM image of a membrane used to sample the L3 drawing: it is possible to distinguish different objects trapped in the polymeric granular structure of the membrane. The backscattered electron detector (BSD) reconstructs a sample image based on the Z of the chemical elements. Therefore, the particles with a higher atomic number appear brighter, and mineral-based shapes could be easily distinguished from the organic ones.

## Discussion

### Art Meets Third Generation Sequencing Technologies

In this study we investigated for the first time some of Leonardo da Vinci’s most emblematic drawings using third generation sequencing technology. One of the most limiting steps found when investigating these invaluable objects, namely the sampling, was carried out using a filtering suction system with small nitrocellulose membranes, which allowed a precise and representative sampling of the total surface of the drawings to obtain all the microbiome on their surfaces.

Furthermore, the tiny nitrocellulose membranes offered the advantage of being easily pooled and used directly for DNA extraction. The total DNA extracted from each drawing was subjected to a metagenomic approach to amplify the entire genomes using the MinION Nanopore sequencing technology. This molecular strategy was already tested by our working group on cultural heritage objects and was shown to be easy for the operator to handle, fast and of a relatively low cost, in comparison with other NGS technologies (Piñar et al., 2020a). The choice of this molecular strategy, including a whole genome amplification protocol (WGA), allowed amplifying all phylogenetic groups at once, enabling to infer the real metagenomes and the relative proportions among the different phylogenetic taxa: eukaryotes, prokaryotes and viruses (Piñar et al., 2020a). An additional advantage of using the Nanopore technology was the length of the sequences generated, which were 4.5 Kb in average, meaning 10-fold longer than with conventional NGS methodologies, as Ion Torrent or Illumina, which yield an average of 400 b length (Kulski, 2016).

### Bacteria vs. Fungi in the Microbiomes of the Drawings

A somewhat surprising result was the higher proportion of bacteria relative to fungi in all the drawings, as fungi were thought to be always dominant in the microbial communities that colonized cultural heritage objects made or supported on paper (Pinzari et al., 2006). Thus, our results could be explained by (1) the sampling strategy used, which was addressed at sampling primarily the dust deposited on the surfaces, and (2) the molecular approach employed in this study.

Concerning the first point, the sampling of visibly biodeteriorated paper often lead to the identification of fungi actively growing and using either cellulose or other compounds. In the case of the Leonardo’s drawings no visible biodeterioration, apart from the foxing stains, could be detected. The high proportion of bacteria in relation to fungi could be an index of the type of deposition and the quality of the storage environment. Bacteria in dust are particularly associated with finer dust particles (Yang J. et al., 2020), which could remain in suspension for longer and even penetrate into the containers where the drawings are stored, and which would then act as a selective filter for certain types of dust. Nevertheless, we cannot rule out that the abundance of bacteria in the drawings investigated in this study may also be due to the source of contamination to which they have been subjected in the recent past, namely human contamination due to restoration works, and therefore with the human skin microbiome, as well as contamination with insect droppings and their gut bacteria.

Regarding the second point, the technologies employed so far could have biased the results in favor of fungi, either through culture-dependent techniques, due to their rapid germination in the appropriate culture medium, or even through molecular techniques. The molecular techniques used so far in paper-based objects, whether of first- (Michaelsen et al., 2006, 2010) or next-generation sequencing (Kraková et al., 2017), have been mainly based on “target amplification” sequencing, which prevents inferring the real proportions of the different target groups (bacteria and fungi). Taking these possible biases into account, this has led to a low number of studies focusing on bacteria colonizing paper materials (Jurado et al., 2010; Michaelsen et al., 2010; Principi et al., 2011; Kraková et al., 2012, 2017). In contrast, there is an extensive literature focused on the colonization of paper by fungi (Sterflinger and Pinzari, 2012). Many fungal species have been described as spoilers of paper-based materials and many of them have shown cellulolytic activities, as some examples species of the genera *Chaetomium*, *Stachybotrys*, *Cladosporium*, *Aspergillus*, *Penicillium.* In this context, one of the main research topics in paper and archival material has been related to the pigments produced by paper colonizing fungi, causing foxing, a deterioration of paper that occurs when brownish and reddish spots are produced by the fungi (Arai, 2000). However, so far, no extensive studies have been conducted to elucidate the total microbiome (bacteria vs. fungi) of these valuable objects. The new technology used in this study has been able to give a new focus to the actual proportions of bacteria/fungi that inhabit paper objects.

### Microbiome Biodiversity in the Drawings

The microbiomes derived from the drawings showed a high biodiversity, with the exception of drawing L4, whose microbiome was heavily contaminated with human sequences, this contamination being perhaps due to its greater handling. In fact, the L4 drawing had massive traces of modern adhesive that required a prolonged conservation work, which was carried out with the aid of a magnification apparatus and various manipulations to which the other drawings were not subjected. This may have resulted in greater removal of surface dust, but at the same time in greater human contamination. Another element to consider in this drawing is the very different nature of its support. The drawing was analyzed with Infrared False Color Photography and Fiber Optics Reflectance Spectroscopy and results showed that the paper was treated with layers of lead white [basic lead carbonate 2PbCO_3_ × Pb(OH)_2_] mixed with indigo (Ruggiero, 2020). Moreover, it appeared that Leonardo followed the recipe described by Cennini and Powell Herringham (1899), which consisted in a preparatory layer made with powdered calcinated chicken bones, white lead, indigo from the plant *Indigofera tinctoria* mixed with animal gelatin and distributed with a brush on the paper support. The design was finally made with a silver tip. This peculiarity may have interfered both with sampling and with the preservation of the microbiome and the environmental DNA in general. Only recent DNA may have remained, i.e., that of the restorers who manipulated it. In this context, Tarsitani et al. (2014) already noted that the period of restoration was probably the critical moment when the discoloration and deterioration of other emblematic drawings by Leonardo da Vinci began, namely in the Atlantic Codex.

#### Bacteria

The results derived from bacteria showed a dominance of *Gammaproteobacteria* of the order *Pseudomonadales*. The genus *Moraxella* was shown to be most abundant at L2, but was also well represented in the L3–L5 drawings, with almost all sequences identified as *M. osloensis*, a saprophytic species in human skin and mucous membranes (Alkhatib et al., 2017). Its high abundance in these drawings can be explained by contamination due to human handling. Nevertheless, this species was also identified in a survey investigating bulls of indulgence from the fifteenth to sixteenth centuries (Jurado et al., 2010). The genus *Acinetobacter* was present in drawings L2, L3, and L7, being most of the sequences related to *A. baumannii.* This species is frequently isolated from hospital environments, although it has been found in environmental samples (Antunes et al., 2014). Nevertheless, sequences related to *Acinetobacter* spp. were previously identified in a cloning and sequencing survey performed on a deteriorated Italian manuscript dated from the thirteenth century (Michaelsen et al., 2010). The genus *Pseudomonas* was relative abundant in drawings L7 and L8, with the dominant species *P. parafulva*, and the genus *Psychrobacter* was representative solely in L5 with most of the sequences affiliating with *Psychrobacter* sp. P11G5, which is a psychrophilic species exhibiting laccase activity (Moghadam et al., 2016). Bacterial laccases (Chauhan et al., 2017) are produced either extracellularly or intracellularly and their activity is in a wide range of temperature and pH. They are different from fungal laccases that are multicopper enzymes belonging to the group of blue oxidases (Janusz et al., 2020).

Members of the *Enterobacteriales* were detected in drawings L2–L6 but interestingly absent in drawings L7–L8. The genus *Salmonella* was detected in L2 and L5, being *Salmonella enterica* subsp. *enterica* serovars identified in both drawings (Vila Nova et al., 2019). In contrast, *Escherichia* was identified in L4–L6, with the species *E. coli*, which is a common inhabitant of the gut microbiota. The genus *Proteus*, with the species *P. mirabilis*, which frequently causes urinary tract infections in humans (Pearson et al., 2008), was present exclusively in L3. Interestingly, two genera identified only in this last drawing, namely *Buchnera* and *Xenorhabdus*, showed the presence of species that are obligate endosymbionts of aphids, such as *B. aphidicola* (Shigenobu and Wilson, 2011) and nematodes, such as *X. bovienii* (Murfin et al., 2015). Interestingly, *X. bovienii* is a bacterium which has been isolated from several nematode *Steinernema* species. *Steinernema* affects many insects and also flies (Archana et al., 2017). Therefore, it is plausible to relate the presence of these species with the presence of insect’s droppings in this drawing.

The genus *Methylovulum* was detected only in drawing L3 and all identified sequences affiliated with *M. psychrotolerans*, a cold-adapted methanotroph from terrestrial environments (Oshkin et al., 2016). Interestingly, the genus *Alcanivorax* was present in drawings L7 and L8, with the dominant species *A. xenomutans*, which has capabilities for alkane degradation and heavy-metal resistance (Fu et al., 2018). Finally, the genus *Stenotrophomonas*, with the species *S. rhizophila* and *S. maltophilia*, was only present in L8 with a high relative abundance. It is important to remark that species of this genus have been identified as colonizers and potential spoilers of oil paintings on canvas support (López-Miras et al., 2013), tempera paintings (Zhgun et al., 2020) as well as ancient paper manuscripts (Michaelsen et al., 2010) and book collections (Kraková et al., 2017; Okpalanozie et al., 2018). In addition, *Stenotrophomonas* sp. has been identified in relation to the biodeterioration of other cultural heritage materials, such as parchments (Piñar et al., 2015a) and historical stone objects (Alakomi et al., 2006; Ettenauer et al., 2011; Rosado et al., 2020). The reason for the perseverance of *Stenotrophomonas* in this type of materials may be due to their extracellular enzyme activities such as proteases and chitinases (Kobayashi et al., 2002) and their capability to produce extracellular polysaccharide, enabling their adhesion to different materials and providing biofilm formation on their surfaces (Alakomi et al., 2006).

The *Betaproteobacteria* were present in all drawings with the dominant genus *Ralstonia*, with species affiliating to *R. solanacearum*, *R. pickettii*, *R. insidiosa*, and *R. mannitolilytica*. *Ralstonia* species, especially *R. pickettii and R. solanacearum* are prevalent colonizers of printing paper machines (Väisänen et al., 1998) and have been detected as dominant species inhabiting damaged oil paints on canvas (Capodicasa et al., 2010; Piñar et al., 2020a). Furthermore, *R. solanacearum* is a phytopathogenic bacterium and may exert an active deterioration on paper materials due to the secretion of extracellular proteins, such as cellulolytic enzymes needed for virulence in plants (Liu et al., 2005). Other *Betaproteobacteria* showed to be relevant only in drawings L7 and L8, as the genera *Burkholderia* and *Cupriavidus*. The *species B. pseudomallei and B. multivorans* were dominant in L7 and *Burkholderia* sp. *MSMB0852 and B*. *mesoacidophila* in L8. Species of this genus were the most frequently isolated from paper products and printing paper machines (Väisänen et al., 1998). *Cupriavidus* sp. are generally heavy metal tolerant bacteria with the ability to degrade a variety of aromatic hydrocarbon compounds (Wang et al., 2015). The species *Cupriavidus* sp. USMAA2-4, *C. metallidurans* and *C. gilardii* were identified in these two drawings.

The *Alphaproteobacteria* class showed to be well represented in drawing L5, with a relative high abundance of species of the genus *Caulobacter*, with the dominance of *C. segnis. Caulobacter* species exert a role in decomposition, including the degradation of cellulose, lignin and polyaromatic hydrocarbons and have the capacity to grow on cellulose (Song et al., 2013). This fact may explain the presence of *Caulobacter* species in this paper-supported drawing and highlights the risk that this bacterium poses to their integrity. This class showed to be relevant also in drawings L7 and L8. The genus *Sphingopyxis* dominated in both drawings. *Sphingopyxis* isolates have been reported to degrade aromatic compounds in various habitats (Yang F. et al., 2020). In addition, the genus *Paracoccus* was only relevant in drawing L7 with the dominance of *P. yeei*, which has been isolated from diverse natural environments, including human skin (Lim et al., 2018). In contrast, the genus *Pannonibacter* was only relevant in L8, being all reads affiliated with *P. phragmitetus*, which is a halotolerant polycyclic aromatic hydrocarbon (PAH)-degrading bacterium (Wang et al., 2016), but it has been also described as a human pathogen (Wang et al., 2017).

The Actinobacteria were the second most represented phylum within the bacteria. The genus *Cryobacterium*, with *C. articum*, showed relatively high abundance in drawings L3–L6. *C. articum* is a psychrotolerant bacterium isolated from extreme cold environments (Bajerski et al., 2011). This species was previously identified in two oil paintings on canvas (Piñar et al., 2020a). The genus *Mycobacterium*, with the dominant species *M. haemophilum*, was present in all the above-mentioned drawings in addition of L7. *M. haemophilum* is an emerging pathogen associated most commonly with skin infections (Tufariello et al., 2015). The genus *Intrasporangium* was present in L2 and L7 with the dominant species *I. calvum*, firstly isolated from the indoor environment (Kalakoutskii et al., 1967). The genus *Nocardia* was identified in drawings L7 and L8 with the dominant species *N. farcinica*. This species causes human infections acquired through the respiratory tract or skin (Torres et al., 2000). The genus *Streptomyces* was also represented in drawings L7 and L8 and in lower proportion in L5. Streptomycetes are ubiquitous in nature and many species are potent producers of lignocellulolytic enzymes including cellulases, hemicellulases and ligninolytic enzymes (Saini et al., 2015). The most dominant species in L7 and L8 was *Streptomyces* sp. CNQ-509, while *Streptomyces albus* dominated in drawing L5. The last species is well-known for its capability to produce xylanases (Saini et al., 2015), which are enzymes responsible for the hydrolysis of β-1,4 bonds in plant xylan, the main component of hemicellulose. The detection of species of this genus in these drawings show a latent risk of deterioration due to the potential cellulolytic activity of these strains. Finally, the genus *Cutibacterium* (*Propionibacterium*) was identified in L3 and L5, with the species *C. acnes* in both drawings. This species is considered as human microbial marker, however, its detection should be considered with caution, as it is a potential pathogen (Fitz-Gibbon et al., 2013).

The phylum Firmicutes was mainly represented by members of the order *Bacillales*, with the dominance of members of the genus *Staphylococcus*, present in L3, L5, L6, and L8 (Figure 6). The dominant species in all mentioned drawings was *S. aureus*, but other species were also identified in high proportions, such as *S. equorum*, *S. capitis*, *S. pasteuri*, and *S. epidermidis*, among others. The genus *Bacillus* was present exclusively in L3, with the dominant species *B. mycoides* and *B. thuringiensis*. Species belonging to *Bacillus* and *Staphylococcus* have been previously identified as the main bacterial species colonizing paper archival materials (Michaelsen et al., 2010; Kraková et al., 2012, 2017; Karakasidou et al., 2018; Okpalanozie et al., 2018).

Members of the order *Lactobacillales* showed to be widely represented in almost all drawings, with the genera *Streptococcus* (in drawings L3–L6), being the emerging pathogen *S. pseudopneumoniae* dominant in L3–L5. This species is associated with lower-respiratory-tract infections and a high prevalence of antimicrobial resistance (Garriss et al., 2019). In contrast, *S. thermophilus* showed to be the dominant species in L6, which is a fermentative lactic acid bacterium (Cui et al., 2016). This species shows acidifying capacity, proteolytic activity, fast growth and production of exopolysaccharide (EPS), with all these capabilities posing a risk to the integrity of the paper support of the drawings. The genus *Lactobacillus* was identified in drawings L5 and L6, with *L. salivarius* and *L. gallinarum* as dominant species in L5 and L6, respectively, which are species frequently isolated from the digestive tract of mammals (Neville and O’Toole, 2010) and chicken. The genus *Lactococcus* was identified in L4, L6, and L7 with *L. piscium* as dominant species in L4 and *L. lactis* in L6 and L7. *L. lactis* has been isolated from a variety of environmental sources but is most commonly associated with fresh or fermented plant material or with milk products (Kelly et al., 2010). *L. piscium* is a psychrotrophic lactic acid bacterium and is known to be one of the predominant species within spoilage microbial communities in cold-stored packaged foods (Andreevskaya et al., 2015). Finally, members of the *Leuconostoc* genus were solely identified in L6. Three species showed to be dominant in this drawing, namely *L. mesenteroides* subsp. *dextranicum*, *L. kimchii*, and *L. citreum*. All of them are commonly used for souring vegetables, producing fermented foods (Dols et al., 1997). However, it is important to note that *Leuconostoc* species have been detected in association with the fruit fly (*Drosophila melanogaster*), whose diet may contain communities of fermentation microbes, including a high percentage of *Leuconostoc* in the fly microbiome (Chandler et al., 2011). Also, the housefly (*Musca domestica* L.) lives in close association with *Leuconostoc* (de Jonge et al., 2020), which was detected mainly in adult flies and associated to the buccal parts of the insects. It is conceivable that the discovery of this bacterium is associated with the presence on the drawings of droppings of insects, mainly related to synanthropic diptera (Jones et al., 1999) and dating back to the time when the drawings were not protected and preserved in an appropriate manner (see section “SEM-EDS”).

Regarding the members of other phyla relatively less abundant in the microbiomes of the drawings, such as *Bacteroidetes*, *Planctomycetes* or *Cyanobacteria* (Figure 4A), it should be mentioned that most of their genera represented less than 0.5% of the total reads. Thus, only the genus *Isosphaera*, with the species *I. pallida* (Planctomycetes phylum) accounted for more than 0.5% of the total reads in L7. Finally, the phylum *Cyanobacteria* was only significantly represented in L3 with species of the genera *Anabaena* (*A. cylindrica*) and *Oscillatoria* (*O. nigro-viridis*), both accounting for more than 0.5% of the total reads in this drawing. Airborne dispersal of microalgae occur at very low concentration in the atmosphere and their occurrence is little known due to technical limitations in investigating microalgae from air samples (Tesson et al., 2016). However, recent studies showed that microalgae can actually be air transported and form a consistent component of airborne biological particulate, depending on the environmental conditions and the local deposition rates (Genitsaris et al., 2011). Furthermore, both the *O. nigro-viridis* and the *A. cylindrica* are cyanobacteria whose tissue can be mineralized with calcium carbonate (Benzerara et al., 2014). Characteristic stromatolite and travertine formations are produced by species precipitating calcium carbonate in their sheaths and within colonial mucilage (Komárek et al., 2003). Some fillers in paper manufacture are based on carbonates from natural sources, such as fossil sedimentary rocks (i.e., biomicrite) (Gerteiser and Laufmann, 1989), which can sometimes be recognized in the form of microfossils among the paper fibers (Bicchieri et al., 2019). It is therefore possible to speculate that the presence of cyanobacteria is due to the use of carbonate-based filler material and natural rocks, perhaps from recent sedimentation, in the manufacture -if not of the Leonardo drawing- perhaps of the cartoons supporting the drawing itself. Both in the case of airborne cyanobacterial propagules and, even more, of biological traces in the sizing minerals, the data confirm the sensitivity of the molecular method used.

It is important to note that it is difficult to compare the data generated in this study with those published in the literature, due to the different technologies applied. As mentioned above, there are not many studies focused on the detection of bacteria on paper-materials and even fewer conducting culture-independent approaches. However, the data generated in this study through third-generation sequencing analyses are somewhat consistent with previous studies where first- and second-generation sequencing was performed. The core microbiome found in this study includes mainly bacteria belonging to the phyla *Proteobacteria*, *Actinobacteria*, and *Firmicutes*, which correlates well with the few previously existing molecular studies. These three phyla were previously detected by different cloning approaches performed on photographic material, including cellulose supports (Bučková et al., 2014; Puškárová et al., 2016) as well as on an ancient Italian manuscript (Michaelsen et al., 2010). Principi et al. (2011) also showed the presence of members of the phyla *Proteobacteria* and *Firmicutes* in the pages of Leonardo da Vinci’s Atlantic Codex. Later, Kraková et al., 2017 confirmed that the core microbiome of various archival materials, investigated with next generation sequencing technology (Illumina MiSeq), consisted mainly of members of the three phyla mentioned above.

To summarize our data, the bacterial species detected in this study can be roughly grouped according to their origin on the paper-support of the drawings. A first group is formed by bacteria that are mainly contaminants introduced by human manipulation, mainly species of the genera *Staphylococcus*, *Streptococcus*, and *Cutibacterium* (*Propionibacterium*), but also species of the genus *Moraxella*, *Paracoccus*, *Mycobacterium*, and *Nocardia* could have been introduced by a mishandling of the drawings. This result is reinforced by a large amount of human DNA found in all the drawings. A second group is formed by bacteria that may have been deposited in the drawings through vectors, such as insects. These bacteria belong to the genera *Salmonella*, *Escherichia*, *Buchnera*, *Xenorhabdus*, and *Leuconostoc*. This result is supported by the finding of insect droppings on the drawings. The third group consists of bacteria that are actually potential spoilers of the paper-support due to their capabilities of producing extracellular enzymes, and they carry a latent risk to the integrity of the drawings. They are members of the genera *Ralstonia*, *Stenotrophomonas*, *Burkholderia*, *Caulobacter*, *Streptomyces*, and *Bacillus*. Some of them could have been introduced into the paper during the production process and others have been deposited on its surface later through the air. However, it is important to note that the bacterial community was influenced by the geographical location of the drawings, Rome or Turin, denoting an environmental influence.

#### Fungi

Among the detected fungi, only some members of the *Ascomycota* phylum represented more than 0.5% of total reads in the microbiomes of the drawings, among them, members of *Sordariomycetes*, *Eurotiomycetes*, *Dothideomycetes*, and *Saccharomycetes*. Within the *Sordariomycetes*, the genus *Colletotrichum* showed to be present in L2, L7–L8. This genus includes several plant pathogens (Cannon et al., 2012). The species *C. higginsianum* was dominant in L7–L8 while *C. orchidophilum* was dominant in L2. Interestingly, the genus *Grosmannia* was only present at L5 in a very high proportion, with *G. clavigera* being the sole species identified. This species is the main fungal pathogen associated with the mountain pine beetle and causes blue stain in wood (Wang et al., 2014). The fungus is linked to American conifers. However, it is important to note that individuals of *Pseudotsuga menziesii* are used as a perennial ornamental conifer species in the northern regions of Italy. It is presumed that the presence of this fungus could be due to a contamination event related to its presence in Rome at the conservation institute where the drawings were analyzed and sampled. In the historical garden of the institute there are in fact ornamental evergreen conifer species. The fungus can also live as a saprotroph in its anamorphic state on bark and cellulosic debris (*Leptographium* form). A further hypothesis is that the drawing came into contact with furniture or woody material, possibly raw, contaminated by the fungus. The DNA in *G. clavigera* mycelium remains stable for more than 10 years also in heat-treated wood samples (Wong et al., 2020). Finally, the genus *Trichoderma* was present in L7 and L8, with the species *T. reesei* as the dominant species in both drawings. *Trichoderma reesei* is one of the main producers of cellulolytic and xylanolytic enzymes, being the most studied fungus involved with lignocellulosic degradation (Bischof et al., 2016). The detection of this fungus supposes a great latent risk of deterioration for these paper-supported drawings if the environmental conditions become suitable for its germination.

Members of the *Eurotiomycetes* were present in drawings L3, L5–L8, being *Aspergillus* widespread in all these drawings, while *Penicillium* showed to dominate in L3 and *Penicilliopsis* in L7. The species *A. glaucus*, *A. clavatus*, and *A. niger* were dominant in all those drawings. *Aspergillus* species are well known for the production of cellulases and their main role in the biodeterioration of paper (Das et al., 1997). The most dominant species found in the drawings, namely *A. glaucus*, is known to have a wide environmental distribution due to its physiological hardiness under extreme conditions and to its ability to grow on substrates with a low water activity (Hubka et al., 2013). *A. glaucus* has been detected in cultural assets made of wooden (Sterflinger et al., 2018b) and canvas support (Piñar et al., 2020a), but its isolation and undistinguishable morphological identification were difficult in the past (Hubka et al., 2013). *Penicillium rubens* showed to be the dominant species in L3. This species is a common fungus of the indoor environment and well known for its resilience under low water activities (van Laarhoven et al., 2017). Finally, *Penicilliopsis zonata* was the dominant members of *Eurotiomycetes* in L7. It is important to note that in the indoor environments, the relative ratio and abundances of fungal species belonging to *Aspergillus*, *Eurotium* and *Penicillium* genera are used along with indicator species to evaluate the presence of hidden moisture damage in buildings (ISO/DIS 16000-17:2006, 2006; Baudisch et al., 2009). Most indoor contamination studies are based on the composition of the dust in suspension. The fungal species in the dust deposited on surfaces are less studied and there are less significant statistics. However, the fact that the drawings, even those preserved in the same environment, presented a different mycoflora is an interesting fact as it allows to formulate hypotheses on the conservation conditions to which the objects have been exposed. The predominance of xerophilic fungal species such as *Penicillium rubens* and *Aspergillus glaucus*, indicates that the drawings have not been preserved in humid environments.

Within the *Dothideomycetes* only members of two genera accounted for more than 0.5% of total reads, namely *Alternaria* and *Bipolaris*. *Alternaria*, with the only detected species *A. alternata* was present in L3 and L5. This fungus is a well-known paper-spoiler (Das et al., 1997; Michaelsen et al., 2006; Mesquita et al., 2009) and also considered as an allergen, imposing a severe health threat on librarians, conservators, and scholars who might be in contact with infected paper-supported materials (Karakasidou et al., 2018). It must be mentioned, however, that the *Alternaria* is a fungus that needs water available in the materials and that it typically develops after flooding and wetting events in building materials, including wood and paper. Therefore, the presence of hydrophilic fungal species on the drawings, in the absence of any visible manifestation of water damage, is due to their common presence in the air as airborne contaminants, also coming from the external environment, from the decomposing plant material typically present in parks and gardens. *Bipolaris sorokiniana* was the main species identified in L7 and L8. *B. sorokiniana* (anamorph of *Cochliobolus sativus*) is a phytopathogenic fungus that produce cell wall-degrading enzymes, such as cellulases, which aid invasion into host cells (Aich et al., 2017). As far as we know, *B. sorokiniana* has not been considered as a paper degrader but, in fact, the fungus is provided with cellulases that can degrade paper fibers and thus, this species poses a latent risk of deterioration for the drawings in which it has been detected.

Members of the *Saccharomycetes*, with *Ascoidea* and *Candida* species were detected in L2–L3 and L5–L6 but curiously, not in drawings L7–L8. *A. rubescens*, *C. orthopsilosis*, and *C. dubliniensis* were the dominant species detected in all four mentioned drawings. *Ascoidea* species appear primarily to be disseminated by insect vectors (Batra, 1963) and have not been identified in paper materials so far. In contrast, *Candida* spp. have been previously identified in archival documents and photographic material, but no evidence regarding implication in paper deterioration has been provided (Principi et al., 2011; Borrego et al., 2015; Karakasidou et al., 2018). The detection and prevalence of species of the genus *Candida* in repositories and archives may be an indication of their ubiquity in this environment, where they can be introduced from the outside environment or contaminated materials after production. Moreover, certain species of *Candida* are typical human microbiota, which might suggest anthropogenic pollution by handling the collections (Borrego et al., 2015, 2017).

In summary, most of the fungi detected in this study have been previously identified in other studies focused on paper-supported materials and include species of the genera *Trichoderma*, *Aspergillus*, *Penicillium*, *Penicilliopsis*, *Alternaria*, and *Candida*. On the contrary, as far as we know, species of other genera, such as *Colletotrichum* and *Bipolaris*, which are mainly plant pathogens, have not been described as common in paper materials. However, they possess extracellular enzymes that may be directly responsible for paper deterioration. Finally, the genera *Grosmannia* and *Ascoidea* include typical species associated with insects and could have been introduced to the surface of the drawings through these vectors. As observed with the bacterial communities, it was also possible to see a correlation of the fungal communities with their geographical location, highlighting an environmental and geographical influence on the microbiomes of the drawings.

### Monitoring of Drawing L2 “*Autoritratto*”

A previous study of the L2 drawing performed in 2012 showed that both the recto and verso had been affected by a fungal attack by the species *Eurotium halophilicum*, a fungus capable of developing under conditions of extremely low substrate water content (<65%). In fact, the fungus is considered as xerophilic and needs hypertonified culture media by adding 15% NaCL for its normal growth, which can also be replaced by other osmoactive organic substances such as sugars (Imai, 1964). In association with the fungal structures, abundant calcium oxalate crystals, resulting from the metabolic activity of the fungus itself, were observed (Pinzari et al., 2014; Piñar et al., 2015b). In 2012 the self-portrait did not show the presence of active infections by microorganisms but only traces of previous attacks and general dust contamination. The membranes and swabs used to sample the dust present on the self-portrait were used to extract the DNA of the microorganisms and to carry out both an evaluation of the type of contamination of the self-portrait (microbiological diversity and abundance) and a comparison, using molecular techniques, of the effectiveness of surface sampling. Details of the study are given in Piñar et al. (2015b), where the authors showed that small membranes are particularly effective for non-invasive sampling from paper and for direct use in DNA extraction protocols. Several fungal species were found to be significantly associated with both the *recto* and the *verso* of the drawing at that time, such as the *Phialosimplex, Penicillium*, and *Acremonium* species. From the molecular analysis, however, it was not possible to obtain gene sequences correlated to the fungus *E. halophilicum*, which was instead diagnosed by SEM analysis, thanks to some unmistakable morphological characters. A possible explanation lies in the treatments that the portrait has undergone over the years. In 1987 Dr. Fausta Gallo, director of the biology laboratory of the former “Istituto Centrale per la Patologia del Libro (Rome)” examined the tracks produced between 1972 and 1987 by the thermo-hydrographers placed in the drawer where the self-portrait was kept. In the spring-summer periods, the portrait was subjected to a Relative Humidity of 70–80% with peaks of 90%. The alarm was such that Dr. Fausta Gallo wrote the same year (1987) that the damage on the portrait was evolving and that disinfection with ethylene oxide was necessary (Pinzari et al., 2014). Ethylene oxide is a disinfectant gas, widely used in the past to sterilize book material, which works as an alkylating agent of nucleic acids and proteins. The chemical action of this compound makes the DNA and RNA of microorganisms (and materials such as parchment) no longer amplifiable (Michaelsen et al., 2013). There is no certainty in the documentation available at ICPAL that the self-portrait has actually undergone disinfection with ethylene oxide. However, if so, any trace of DNA present on the object and prior to treatment would be undetectable with current analysis techniques. In this case, the infection by the fungus *E. halophilicum*, evident in the images obtained with SEM but not detected either by molecular techniques based on cloning in 2012, and currently by nanopore technology, would be dated back to before 1987. The design has not been treated in any way since 2012. The new series of molecular analyses, carried out by means of Nanopore sequencing, a much more advanced technique than cloning, should have detected the presence of the fungus, which instead did not emerge. Other typical air contaminating species were detected, despite their much lower density than that documented for the *E. halophilicum*. Therefore, the absence of the fungal species among those found with Nanopore sequencing supports the hypothesis that the design was actually treated with ethylene oxide in 1987.

### SEM-EDX

The incrustations analyzed so far were found to be insect droppings (Figure 8). This evidence is due partly to the presence of spherical particles of biogenic nature composed of magnesium and calcium phosphate recognized as “spherulites” which are biogenic stones that form in the gut of insects or in their excretion products (Gower, 2008). These spherical micro-objects are used in archeology as markers of the presence or use of animals’ dung (Lancelotti and Madella, 2012). The other encrustations, those without spherulites, appeared to be instead rich in nitrogen compounds, and potassium sulfate, that are both catabolites of insects’ metabolism (Cochran, 1975). The small monoclinic and prismatic crystals characterizing these objects were scarcely distinguishable in the matrix when visualized with BSD detector, which means that they were not presenting a high atomic number, and could presumably be uric salts (uric acid: C_5_H_4_N_4_O_3_). Nitrogen could make cellulose potentially more degradable since nitrogen-based compounds are substances normally absent in paper media, but which microorganisms need to grow (Pinzari, 2011). The insects’ droppings found on the L4 drawing were colonized by fungal mycelium. Leonardo’s drawings have been kept for many years locked in vaults, protected by cardboard boxes and other protective systems. To imagine that an insect can rest on a drawing to leave its droppings is unrealistic. Therefore, the waxy incrustations removed and analyzed could date back for many years. That is, when the drawings were exposed to the air, so even before they were hung in glass frames, as some appear to have been around the 1930s and 1950s (Misiti, 2014). The species of insects that cause damage to books and libraries belong to different families, united by having representatives who have adapted to living in man-made environments and have spread throughout the world thanks to commercial activities. According to their biological characteristics, they carry out their life cycle within the materials they feed on or in the surrounding environment (Florian, 1997). In the case of the traces found on the drawings, rather than the classic insects harmful to paper and parchment, which typically cause lacunas and tracks such as cockroaches, woodworms and silverfishes, it seems that the droppings are more characteristic of species of diptera such as domestic flies and related species. House flies (*Musca domestica*) are synanthropic and commonly found on decaying matter but also feces and food. They have a complex prokaryotic microbiota in their gut, that they can release on materials through droppings and regurgitation of food. Furthermore, the microbiome in domestic flies can be relatively consistent across geographic locations and habitat (Park et al., 2019).

Finally, we would like to make a general discussion about the conservation over the centuries of these unique and very fragile objects, in terms of the need to remove insect excrements, dust and the remains of fungi and bacteria, since they are all substances rich in nitrogen, and chemically reactive compounds, capable of oxidizing and degrading the paper fibers. Microorganisms that are still viable also pose other risks, as they can proliferate and cause much more serious damage under certain environmental conditions. However, the manipulation of the drawings could also lead to possible additional contamination, as demonstrated in this study by the presence of bacteria from the human microbiome and the abundant human DNA. However, thanks to the “dirt” found on these heritage objects it has been possible to study their history and to draw a distinctive profile that forms part of the drawings themselves and describes them. The information obtained in this study comes from samples that would be eliminated in a routine conservative treatment. Incrustations, amorphous particles, pieces of glue and remains of supporting cardboard are materials that have been removed by restorers up to now. However, in light of what can be discovered from their meticulous study, the process of conserving certain artistic objects probably needs to be reconsidered because part of their history is written in their “dirt”.

## Conclusion

This study shows for the first time the complete microbiomes of some of Leonardo da Vinci’s most emblematic drawings. The strategy applied for their research, namely the Nanopore sequencing technology, is one of the most advanced so far to investigate all the phylogenetic groups included in each of the microbiomes. In addition, the complementation with SEM analysis allowed the visualization of insect droppings in some of the drawings, which are directly correlated with many of the microorganisms found on their surfaces.

The results show a surprising dominance of bacteria over fungi. This fact is partly due to the sampling strategy used, aimed at sampling mainly the dust deposited on the surface of the drawings, but also to the source of contamination to which they have been subjected in the recent past, resulting in a high proportion of bacteria typical of the human microbiome, introduced by intensive handling of the drawings during restoration works, in addition to other microorganisms that have obviously been introduced as contaminants through vectors, such as insects and their excrements.

It is important to clarify that, unlike other case studies that point to visible paper damage caused by fungi, often also associated with bacteria, no clear damage apart from foxing was observed in this study. The sensitivity of the Nanopore sequencing method, which provides information on the abundance of the different taxa in each sample, could have an advantage over other methods for monitoring an ongoing deterioration process, because it allows the assessment of quantitative relationships between taxa and also the deduction of variations in these relationships due to detrimental situations.

In general, the biological information contained in the investigated drawings showed very specific microbiomes, which can be used as a bio-archive of the history the objects, providing a kind of fingerprint for current and future comparisons. However, some similarities were observed that could be influenced by the geographical location of the drawings. Some taxa were only detected in the drawings stored in the Royal Library of Turin, while other taxa were only detected in the drawings stored in the Corsinian Library in Rome.

The results show a high proportion of human DNA, especially in drawing L4, which indicates an inadequate manipulation of this drawing, most probably during the previous restoration processes. This result indicates that restoration techniques should be continually reviewed based on scientific evidence and that monitoring studies in the field of conservation and restoration should be encouraged.

Finally, in order to maximize the value of the samples that can be obtained from unique artworks, it is critical to optimize the analytical processes through protocols that allow the conservation and reuse of the samples in various analyses. Analytical techniques based on the study of environmental DNA will enable the maximum exploitation of natural bio-archives represented by dust, biofilm or other traces of past events present in the works. In perspective, it is possible to imagine a pipeline of measures aimed at obtaining the maximum information from samples taken from unique objects, such as Leonardo’s drawings, which allow very little sampling. Measuring the vitality and activity of individual microbial cells present in materials is probably the next challenge. There are techniques that have already been consolidated in other fields of biology, such as cytofluorimetric measures and single-cell RNA sequencing, which examines the sequence information of individual cells with optimized NGS technologies, which would allow a better understanding of the function of an individual microorganism in the context of its microenvironment. In the light of the new research techniques and their resolving power, some considerations on the impact that cleaning and consolidation techniques may have on the information content of ancient materials are now really urgent. Likewise, new methods and protocols are needed, both for the documentation and for the recovery of these natural “bio-archives” that accompany all the unique objects of our artistic and cultural heritage, without weighing down the already arduous work of conservators and restorers.

## Data Availability Statement

The datasets presented in this study can be found in online repositories. The names of the repository/repositories and accession number(s) can be found below: https://www.ncbi.nlm.nih.gov/, Bioproject PRJNA655381.

## Author Contributions

GP conceived the design of the study, conducted molecular analyses, processed sequencing data, and wrote the manuscript. MSc and PC conducted sampling and participated in SEM analyses. FP performed and analyzed SEM and SEM-EDS and contributed to the writing of the manuscript. AG analyzed the sequencing data and contributed to the writing of the manuscript. MSe and KS conceived and contributed to the design of the study and supervised the project. All authors approved the final version for submission.

## Conflict of Interest

The authors declare that the research was conducted in the absence of any commercial or financial relationships that could be construed as a potential conflict of interest.
