# Supplementary material for: The Microbiome of Leonardo da Vinci’s Drawings: A Bio-Archive of Their History
Source: Front Microbiol. 2020 Nov 20;11:593401. doi: 10.3389/fmicb.2020.593401 (PMC7718017; doi:10.3389/fmicb.2020.593401)
Supplement: Supplementary file 1 [file Presentation_1.PPTX]

## Slide 1
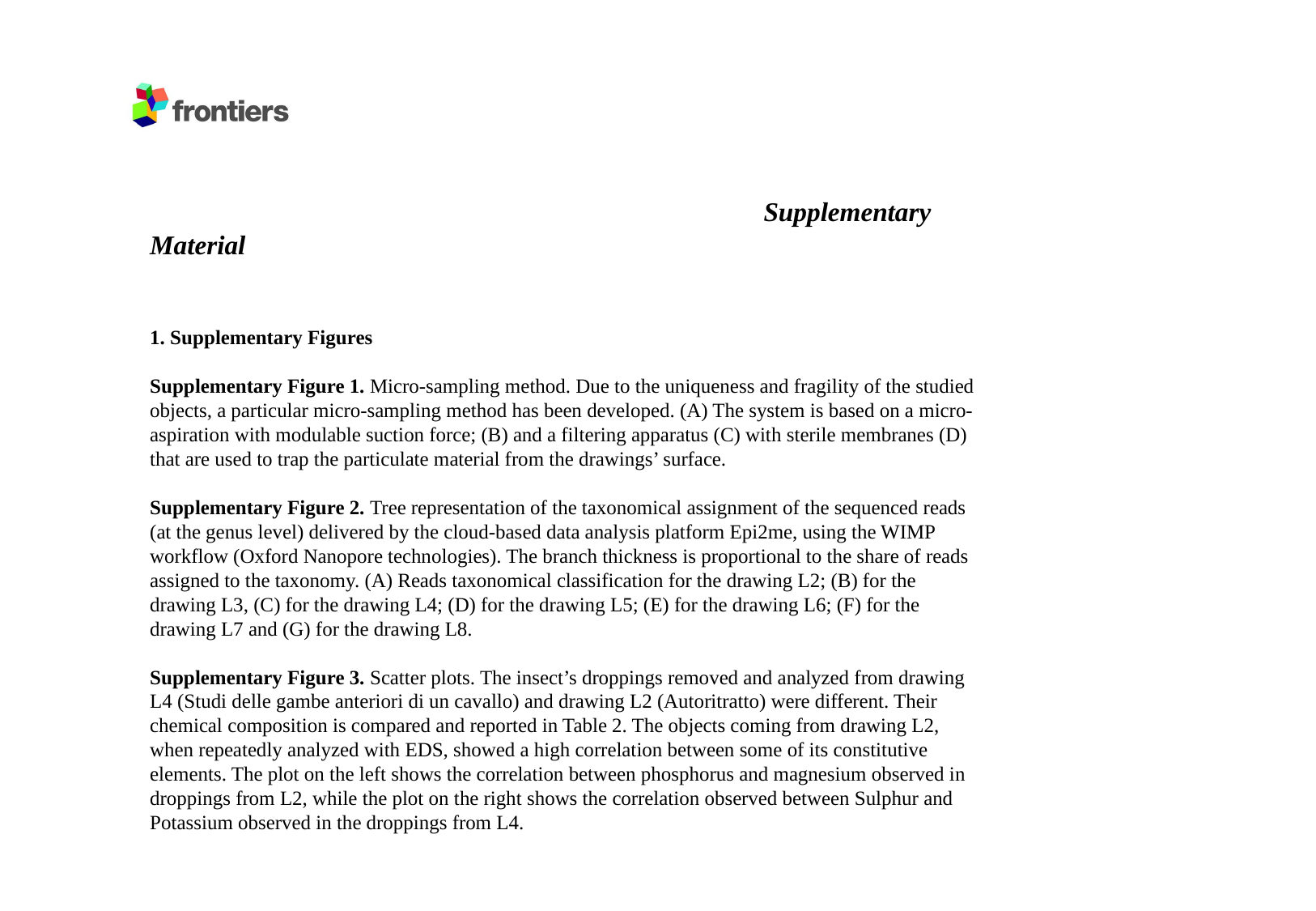

Supplementary Material
1. Supplementary Figures
Supplementary Figure 1. Micro-sampling method. Due to the uniqueness and fragility of the studied objects, a particular micro-sampling method has been developed. (A) The system is based on a micro-aspiration with modulable suction force; (B) and a filtering apparatus (C) with sterile membranes (D) that are used to trap the particulate material from the drawings’ surface.
Supplementary Figure 2. Tree representation of the taxonomical assignment of the sequenced reads (at the genus level) delivered by the cloud-based data analysis platform Epi2me, using the WIMP workflow (Oxford Nanopore technologies). The branch thickness is proportional to the share of reads assigned to the taxonomy. (A) Reads taxonomical classification for the drawing L2; (B) for the drawing L3, (C) for the drawing L4; (D) for the drawing L5; (E) for the drawing L6; (F) for the drawing L7 and (G) for the drawing L8.
Supplementary Figure 3. Scatter plots. The insect’s droppings removed and analyzed from drawing L4 (Studi delle gambe anteriori di un cavallo) and drawing L2 (Autoritratto) were different. Their chemical composition is compared and reported in Table 2. The objects coming from drawing L2, when repeatedly analyzed with EDS, showed a high correlation between some of its constitutive elements. The plot on the left shows the correlation between phosphorus and magnesium observed in droppings from L2, while the plot on the right shows the correlation observed between Sulphur and Potassium observed in the droppings from L4.

## Slide 2
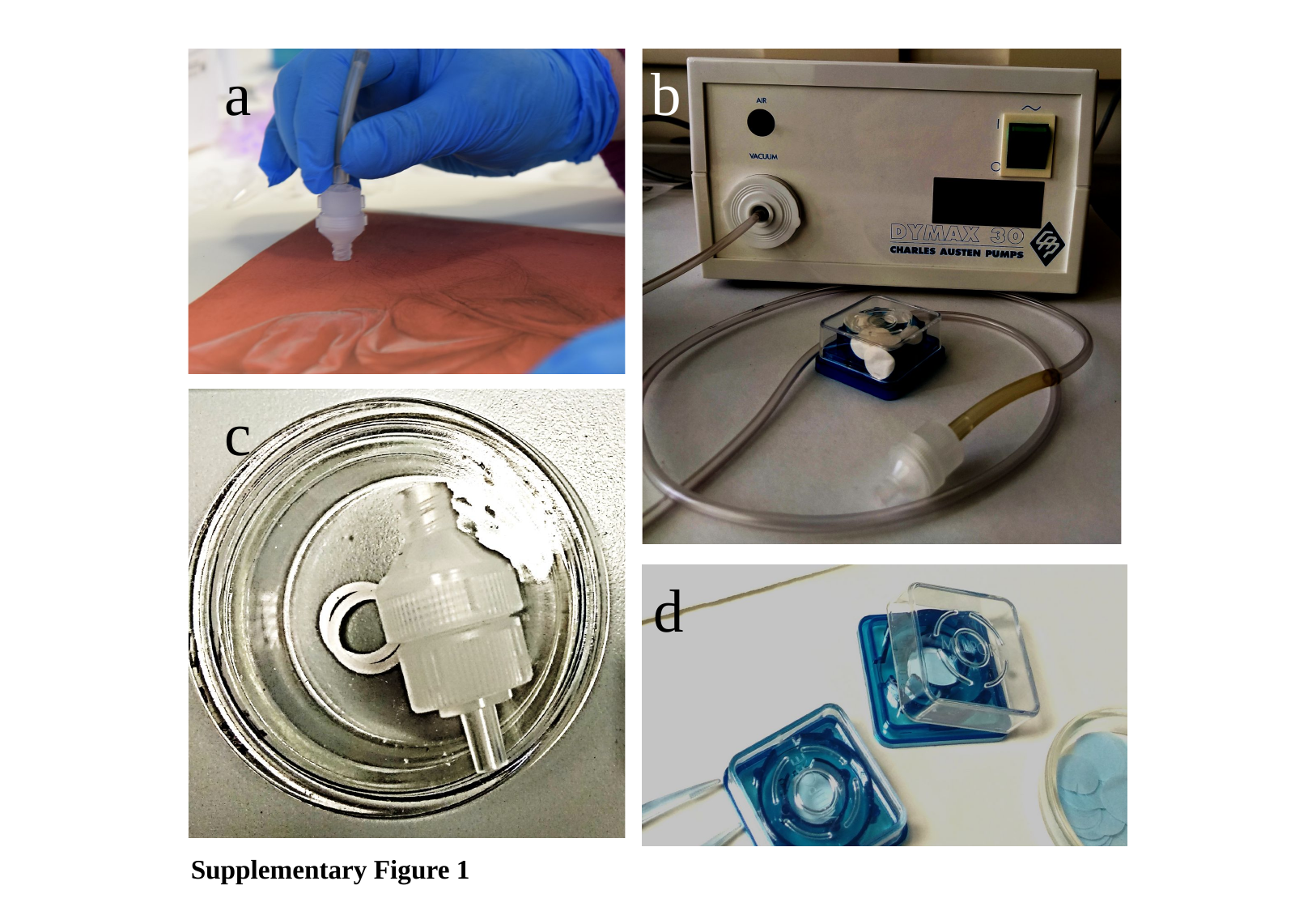

b
a
c
d
Supplementary Figure 1

## Slide 3
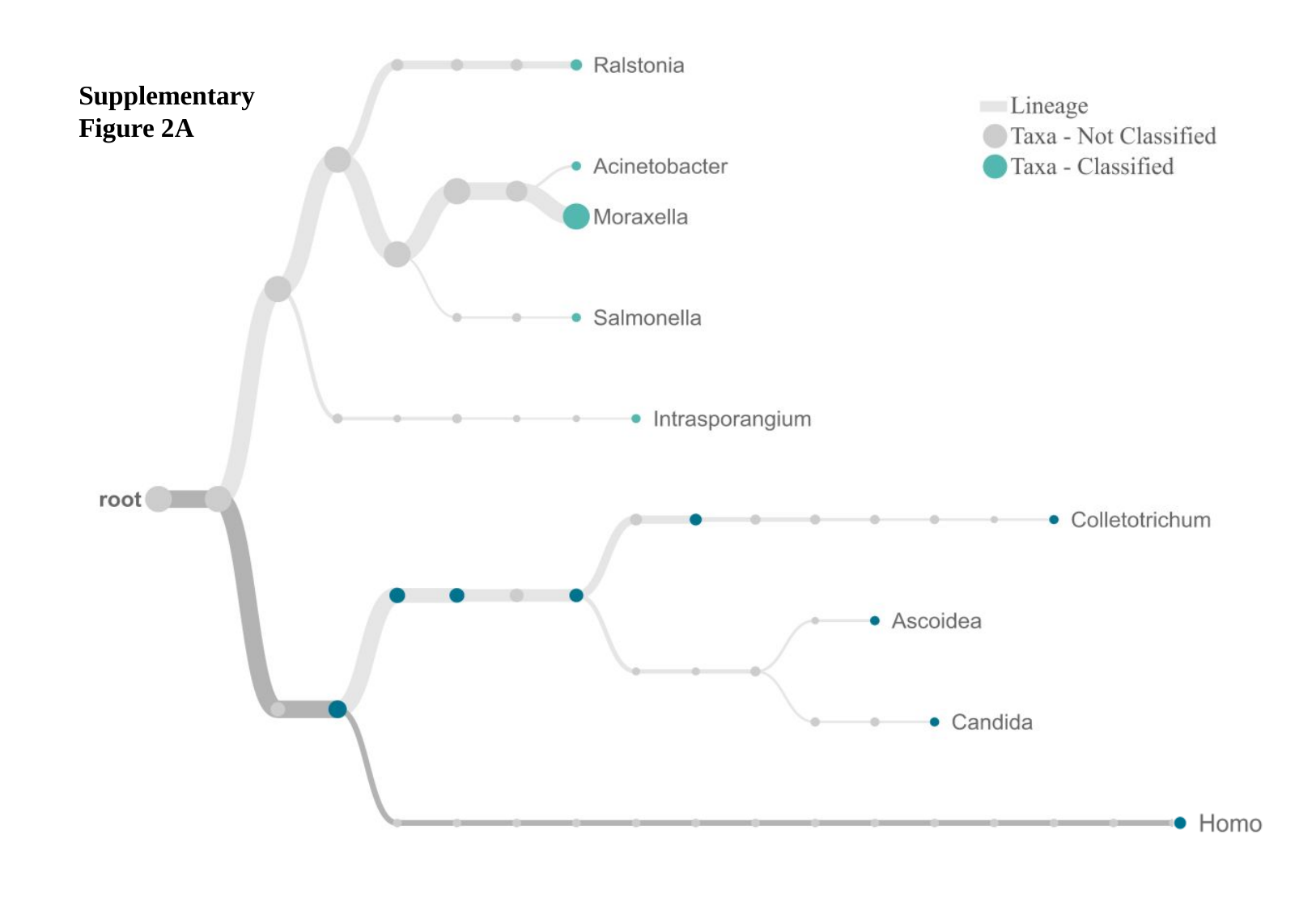

Supplementary
Figure 2A

## Slide 4
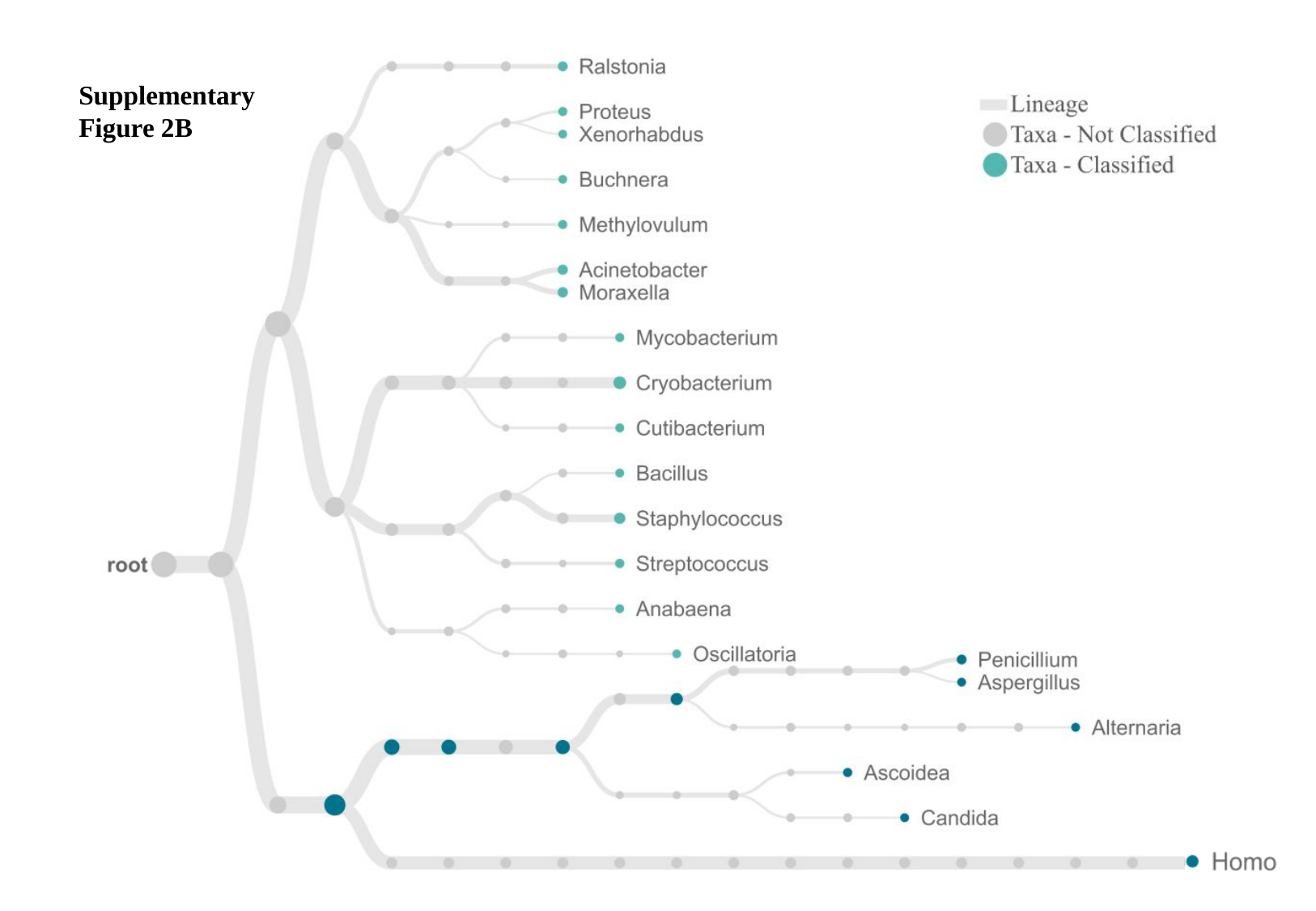

Supplementary
Figure 2B

## Slide 5
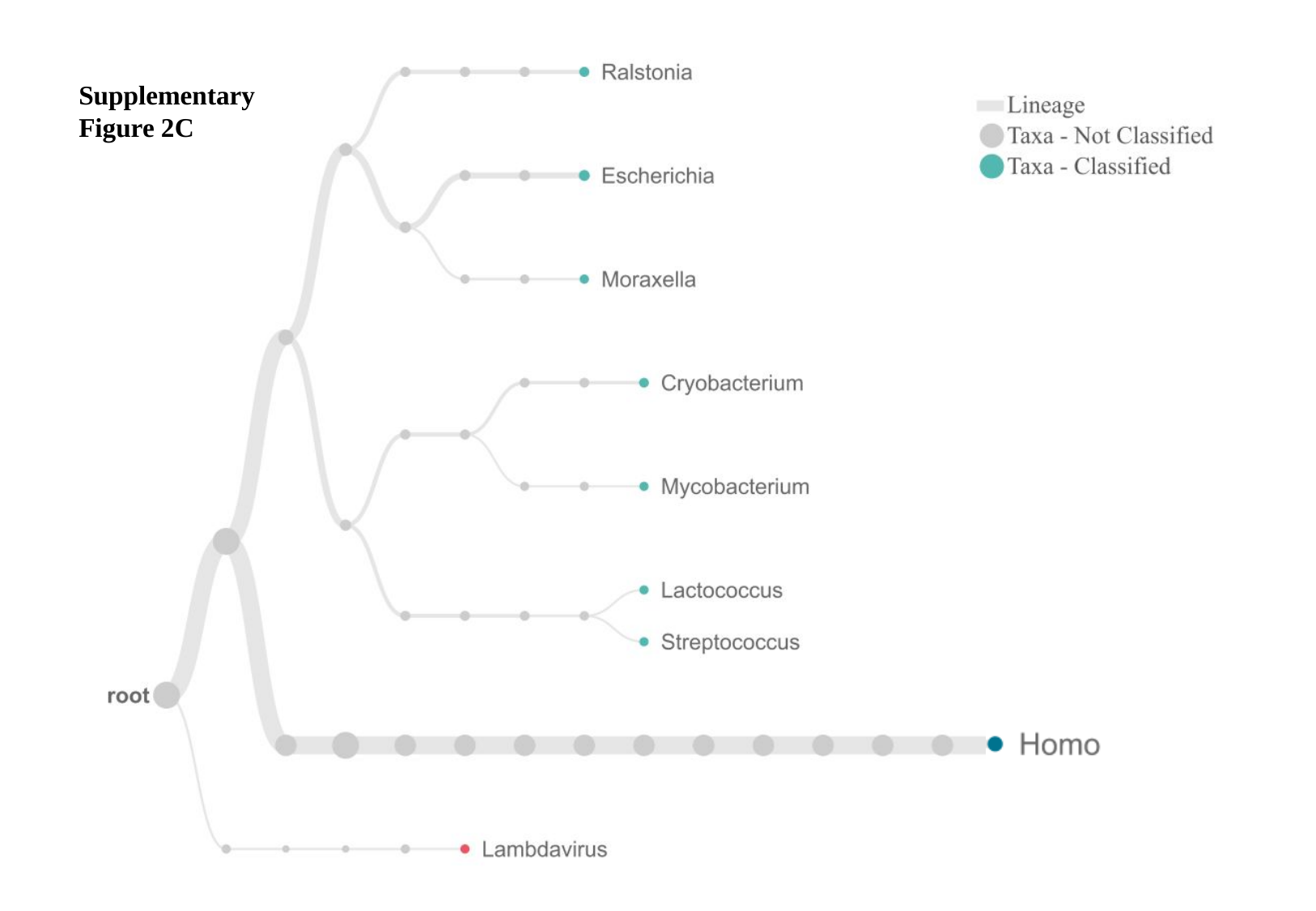

Supplementary
Figure 2C

## Slide 6
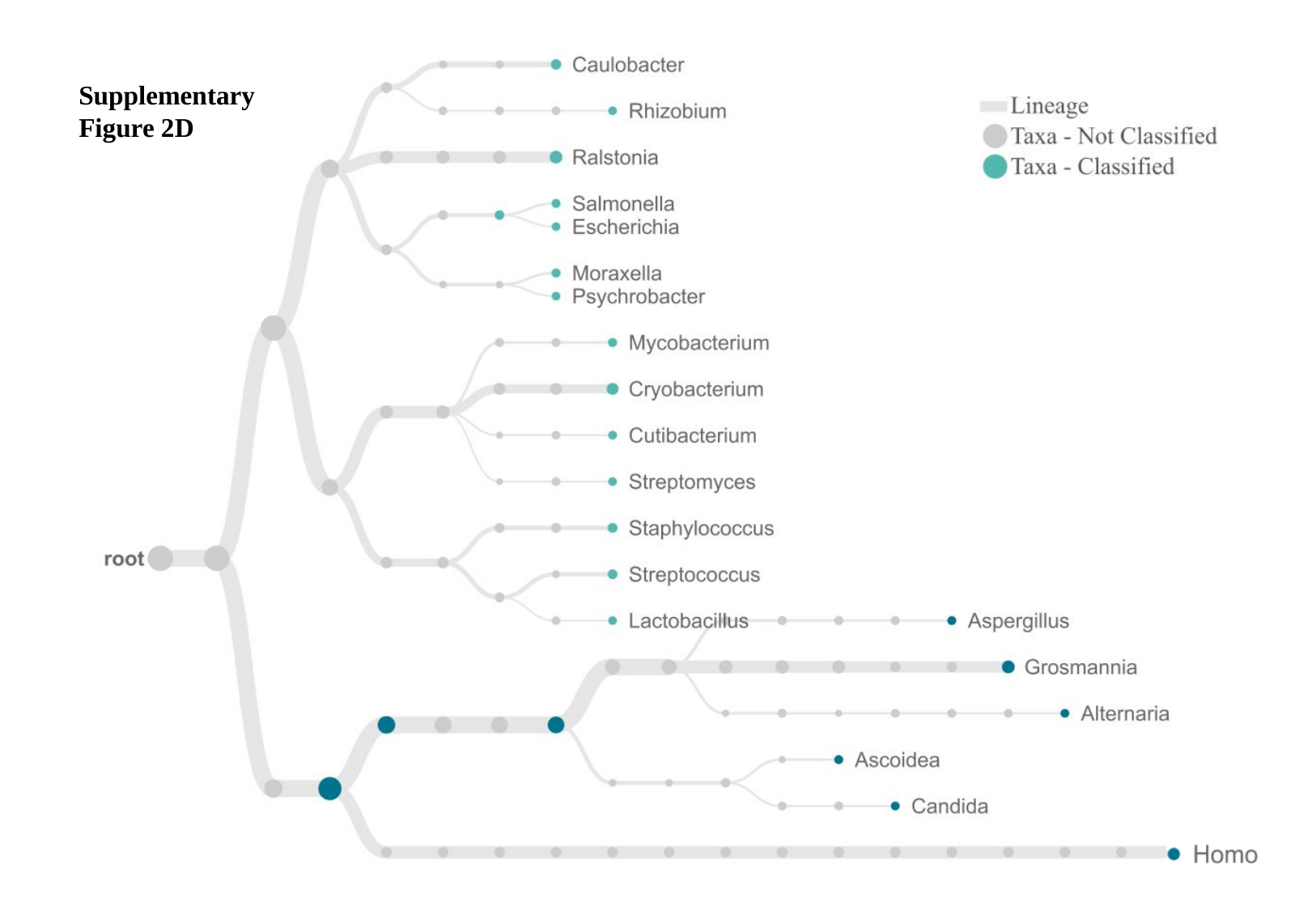

Supplementary
Figure 2D

## Slide 7
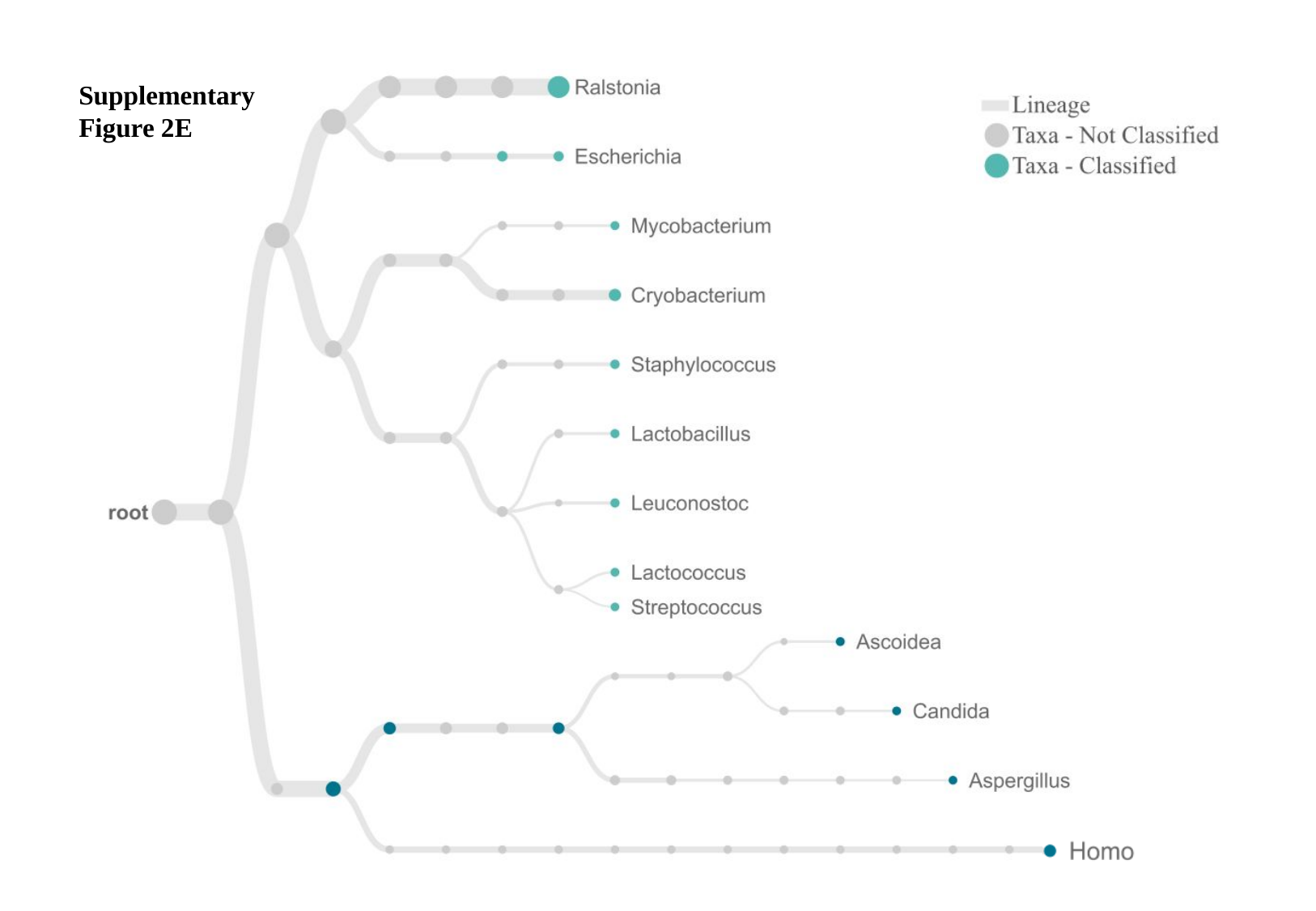

Supplementary
Figure 2E

## Slide 8
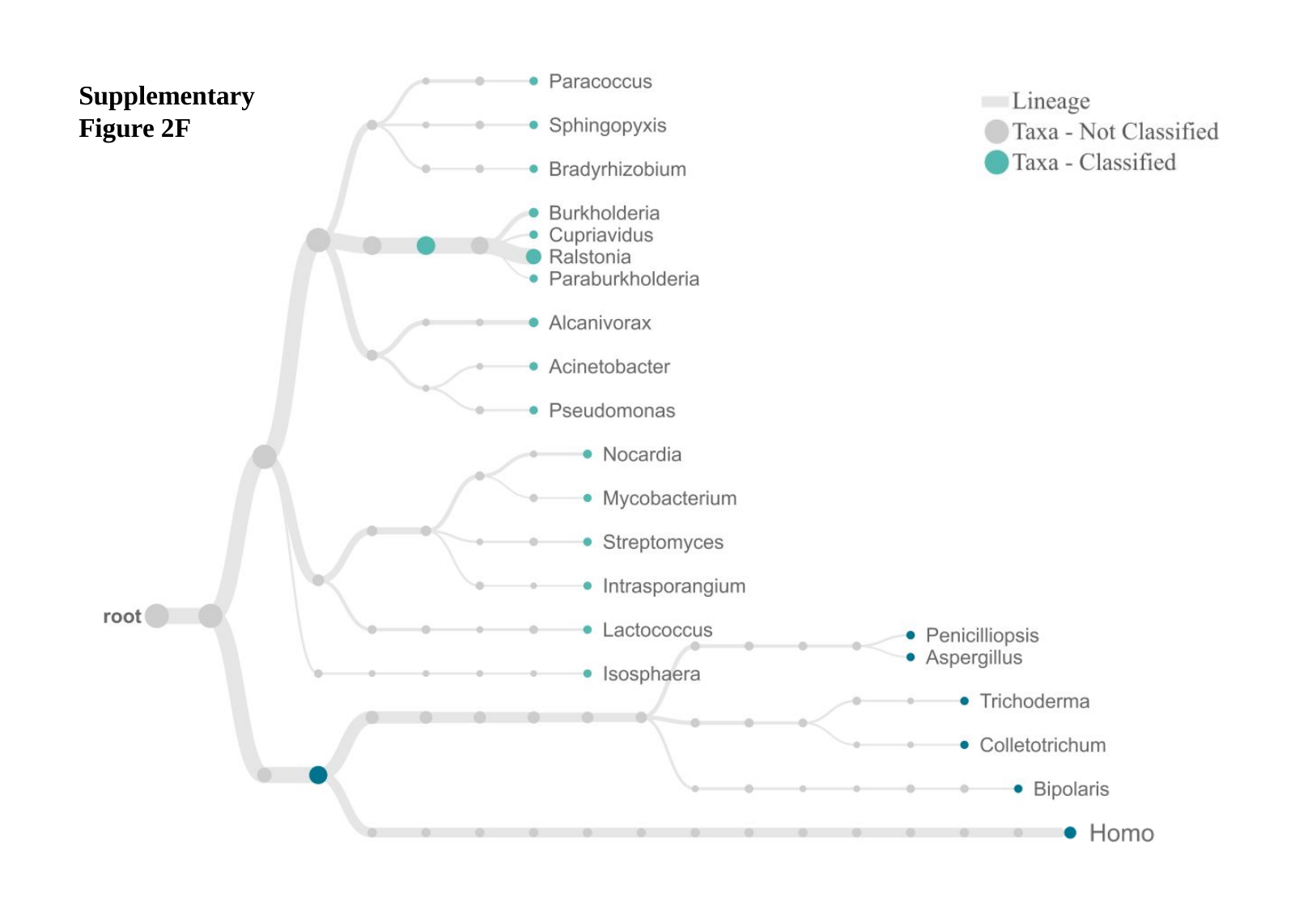

Supplementary
Figure 2F

## Slide 9
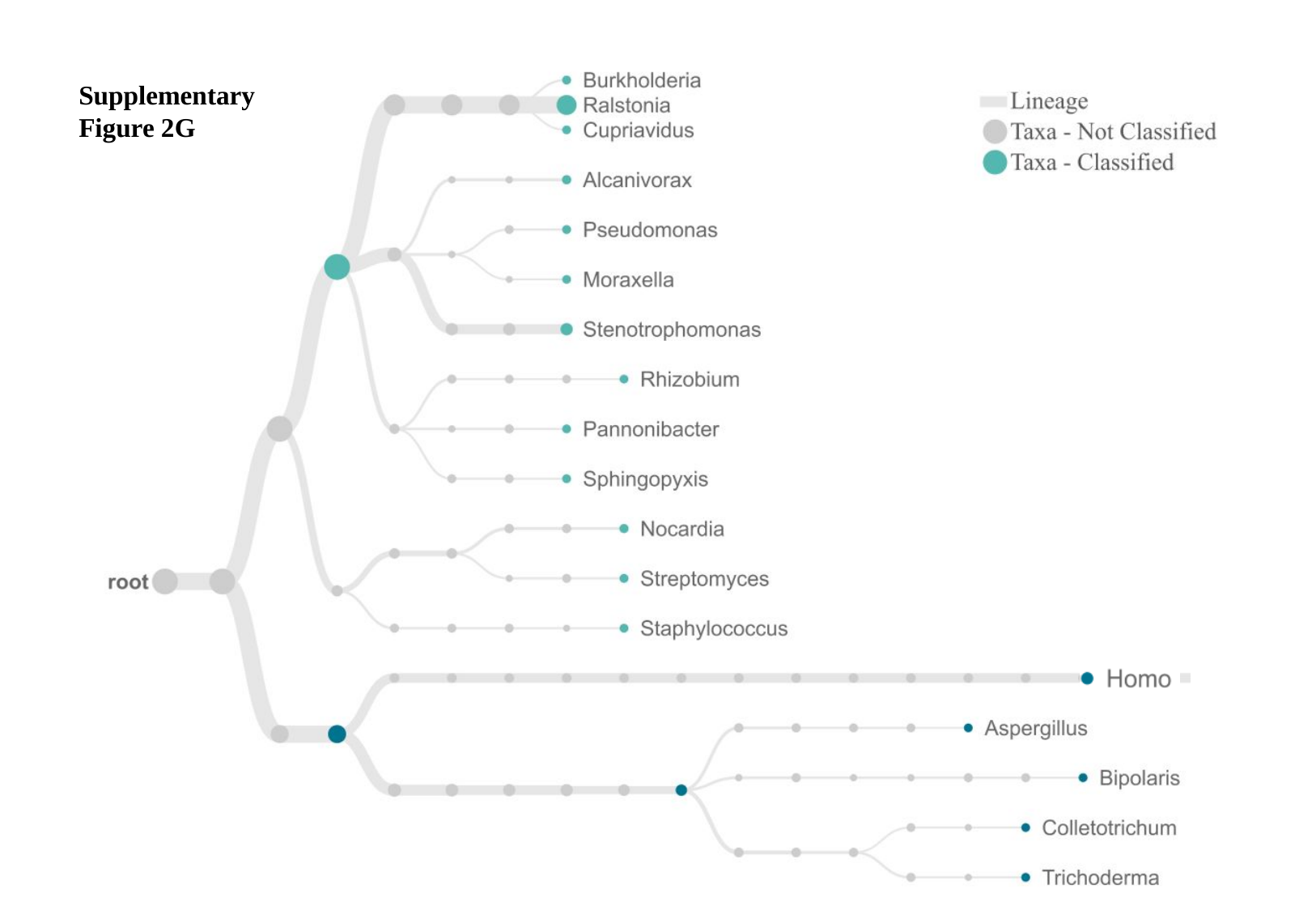

Supplementary
Figure 2G

## Slide 10
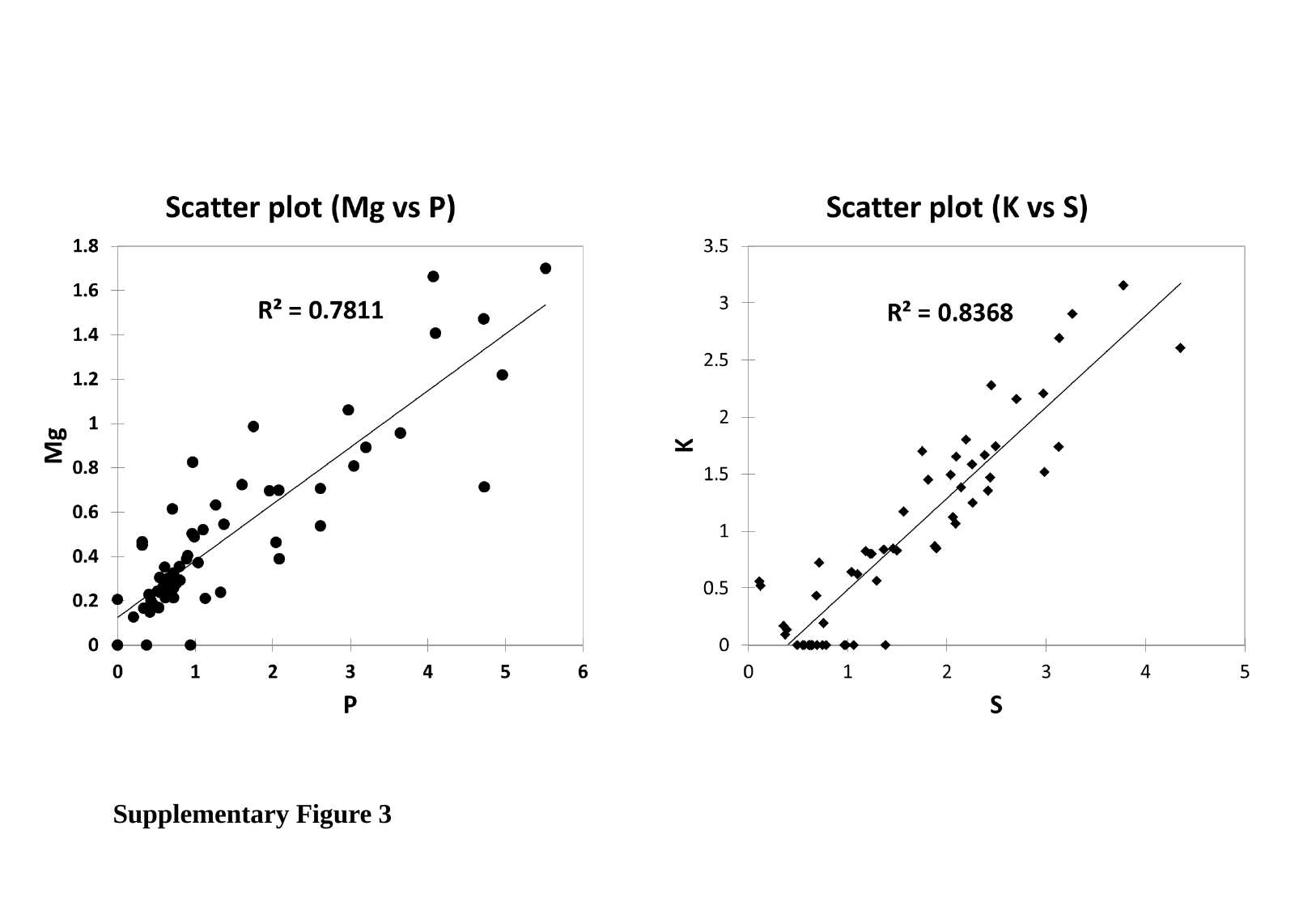

Supplementary Figure 3
